# A Model System for Studying the Transcriptomic and Physiological Changes Associated with Mammalian Host-Adaptation by *Leptospira interrogans* Serovar Copenhageni

**DOI:** 10.1371/journal.ppat.1004004

**Published:** 2014-03-13

**Authors:** Melissa J. Caimano, Sathesh K. Sivasankaran, Anna Allard, Daniel Hurley, Karsten Hokamp, André A. Grassmann, Jay C. D. Hinton, Jarlath E. Nally

**Affiliations:** 1 Department of Medicine, University of Connecticut Health Center, Farmington, Connecticut, United States of America; 2 Department of Pediatrics, University of Connecticut Health Center, Farmington, Connecticut, United States of America; 3 Department of Microbiology, School of Genetics and Microbiology, Moyne Institute of Preventative Medicine, Trinity College Dublin, Dublin, Ireland; 4 School of Veterinary Medicine, University College Dublin, Belfield, Dublin, Ireland; 5 Department of Genetics, School of Genetics and Microbiology, Smurfit Institute of Genetics, Trinity College Dublin, Dublin, Ireland; 6 Unidade de Biotecnologia, Centro de Desenvolvimento Tecnológico, Universidade Federal de Pelotas, Pelotas, Rio Grande do Sul, Brazil; 7 Department of Functional and Comparative Genomics, Institute of Integrative Biology, University of Liverpool, Liverpool, United Kingdom; 8 Conway Institute of Biomolecular and Biomedical Research, University College Dublin, Belfield, Dublin, Ireland; Medical College of Wisconsin, United States of America

## Abstract

Leptospirosis, an emerging zoonotic disease with worldwide distribution, is caused by spirochetes belonging to the genus *Leptospira*. More than 500,000 cases of severe leptospirosis are reported annually, with >10% of these being fatal. Leptospires can survive for weeks in suitably moist conditions before encountering a new host. Reservoir hosts, typically rodents, exhibit little to no signs of disease but shed large numbers of organisms in their urine. Transmission occurs when mucosal surfaces or abraded skin come into contact with infected urine or urine-contaminated water or soil. In humans, leptospires can cause a variety of clinical manifestations, ranging from asymptomatic or mild fever to severe icteric (Weil's) disease and pulmonary haemorrhage. Currently, little is known about how *Leptospira* persist within a reservoir host. Prior *in vitro* studies have suggested that leptospires alter their transcriptomic and proteomic profiles in response to environmental signals encountered during mammalian infection. However, no study has examined gene expression by leptospires within a mammalian host-adapted state. To obtain a more faithful representation of how leptospires respond to host-derived signals, we used RNA-Seq to compare the transcriptome of *L. interrogans* cultivated within dialysis membrane chambers (DMCs) implanted into the peritoneal cavities of rats with that of organisms grown *in vitro*. In addition to determining the relative expression levels of “core” housekeeping genes under both growth conditions, we identified 166 genes that are differentially-expressed by *L. interrogans in vivo*. Our analyses highlight physiological aspects of host adaptation by leptospires relating to heme uptake and utilization. We also identified 11 novel non-coding transcripts that are candidate small regulatory RNAs. The DMC model provides a facile system for studying the transcriptional and antigenic changes associated with mammalian host-adaption, selection of targets for mutagenesis, and the identification of previously unrecognized virulence determinants.

## Author Summary

Leptospirosis, a global disease caused by the unusual bacterium *Leptospira*, is transmitted from animals to humans. Pathogenic species of *Leptospira* are excreted in urine from infected animals and can continue to survive in suitable environments before coming into contact with a new reservoir or accidental host. Leptospires have an inherent ability to survive a wide range of conditions encountered in nature during transmission and within mammals. However, we know very little about the regulatory pathways and gene products that promote mammalian host adaptation and enable leptospires to establish infection. In this study, we used a novel system whereby leptospires are cultivated in dialysis membrane chambers implanted into the peritoneal cavities of rats to compare the gene expression profiles of mammalian host-adapted and *in vitro*-cultivated organisms. In addition to providing a facile system for studying the transcriptional and physiologic changes leptospires undergo during mammalian infection, our data provide a rational basis for selecting new targets for mutagenesis.

## Introduction

Leptospirosis is a neglected disease of global significance [1], [2]. Pathogenic leptospires, shed in animal urine or free-living within contaminated water, enter the host through small abrasions in the skin or contact with mucous membranes of the eyes, nose or throat. Organisms disseminate almost immediately following acquisition, travelling *via* the bloodstream to multiple tissues [3]. *L. interrogans*, an extracellular pathogen, is thought to penetrate host tissues by intercellular migration [4]. In immunocompetent hosts, the majority of leptospires are thought to be cleared by opsonophagocytosis following the appearance of specific antibodies [5]. However, organisms that reach the kidneys, an immunoprivileged site [1], adhere to and colonize the proximal convoluted renal tubules, where they replicate exponentially. The majority of human disease is caused by *Leptospira interrogans* serovar (sv.) Copenhageni for which *Rattus norvegicus* serves as a reservoir host [3], [6], [7]. Experimentally-infected rats can excrete up to 10^7^ leptospires/ml of urine for months without clinical signs of infection, thus exemplifying the unique biological equilibrium that can exist between pathogen and reservoir host [8], [9], [10].

The genome sequences of several pathogenic and saprophytic *Leptospira* spp., including *L. interrogans* sv. Copenhageni, are now complete [6], [11], [12], [13], [14], [15], [16]. *L. interrogans* sv. Copenhageni Fiocruz L1-130 harbors 3728 protein-encoding genes [11], [12]. By comparative genomics, Picardeau *et al.* [14] identified 1431 “pathogen-specific” genes that are present within either or both of the pathogenic species, *L. interrogans* and *L. borgpetersenii*, but are absent from the free-living saprophyte *L. biflexa*. Although the majority (62%) of these pathogen-specific genes encode proteins of unknown function, it is possible that some are required by *Leptospira* to respond to unique environmental cues encountered within the mammalian host. Along these lines, the genome of *L. interrogans* contains >200 protein-coding sequences potentially involved in gene regulation, including gene products associated with two component signal transduction systems, alternate sigma factors, anti-sigma factors, and anti-sigma factor antagonists [11], [12]. Not surprisingly, the pathogen-specific group also includes numerous gene products whose annotated functions or cellular location suggest a potential role in virulence-related processes such as adherence, digestion of host tissues and extracellular matrix, and evading the host's innate and adaptive immune responses [14], [17].

To identify novel leptospiral virulence determinants, investigators have manipulated *in vitro* growth conditions to simulate those encountered within the mammalian host, including increased temperature and/or osmolarity, iron starvation, and the presence of serum [18], [19], [20], [21], [22]. However, the extent to which these *in vitro* conditions faithfully reproduce those encountered by *Leptospira in vivo* is unclear. In an effort to characterize leptospires in a truly mammalian host-adapted state, we cultivated virulent low-passage *L. interrogans* sv. Copenhageni within the peritoneal cavities of rats using a modification of our dialysis membrane chamber (DMC) model [23], [24]. Given that rats are a natural reservoir host for this species of *Leptospira* [2], [25], [26], we reasoned that this model would be ideal for this purpose. Originally developed to study host adaption by Lyme disease spirochetes (*Borrelia burgdorferi*) [23], [24], this technique, which uses dialysis membrane tubing with an 8000 Da molecular weight cut-off, provides bacteria with access to host nutrients while protecting them from the host's cellular immune response. The DMC model has been instrumental in studying the contribution of mammalian host-specific signals to differential gene expression in *B. burgdorferi* on a genome-wide scale as well as enabling us to characterize the transcriptional and physiological changes integral to the mammalian host-adaptation process [23], [24], [27], [28], [29].

In recent years, high-throughput RNA sequencing (RNA-Seq) has replaced microarrays as the method of choice for genome-wide transcriptional profiling in bacteria [30], [31]. Unlike microarrays, RNA-Seq allows transcription to be understood at the single-nucleotide level. Here, we used an RNA-Seq approach to compare the transcriptome of virulent low passage *Leptospira interrogans* sv. Copenhageni cultivated within DMCs with that of leptospires grown under standard *in vitro* conditions (30°C in EMJH). Using this approach, we determined the relative expression levels of “core” housekeeping genes under both growth conditions, and, more importantly, we identified 166 genes that are differentially-expressed by leptospires within the mammalian host, the majority of which are pathogen-specific (*i.e.*, not present within saprophytic *Leptospira*). Most notably, our analyses highlight novel physiological aspects of mammalian-host adaptation by leptospires with respect to heme uptake and utilization. Moreover, we identified 11 novel non-coding (ncRNAs) transcripts which represent candidate small regulatory RNAs. In addition to providing a facile system for studying the transcriptional and physiologic changes leptospires undergo during mammalian infection, our data provide a rational basis for selecting new targets for mutagenesis.

## Results

### Virulent leptospires become mammalian host-adapted during growth within dialysis membrane chambers

**Figure 1:**
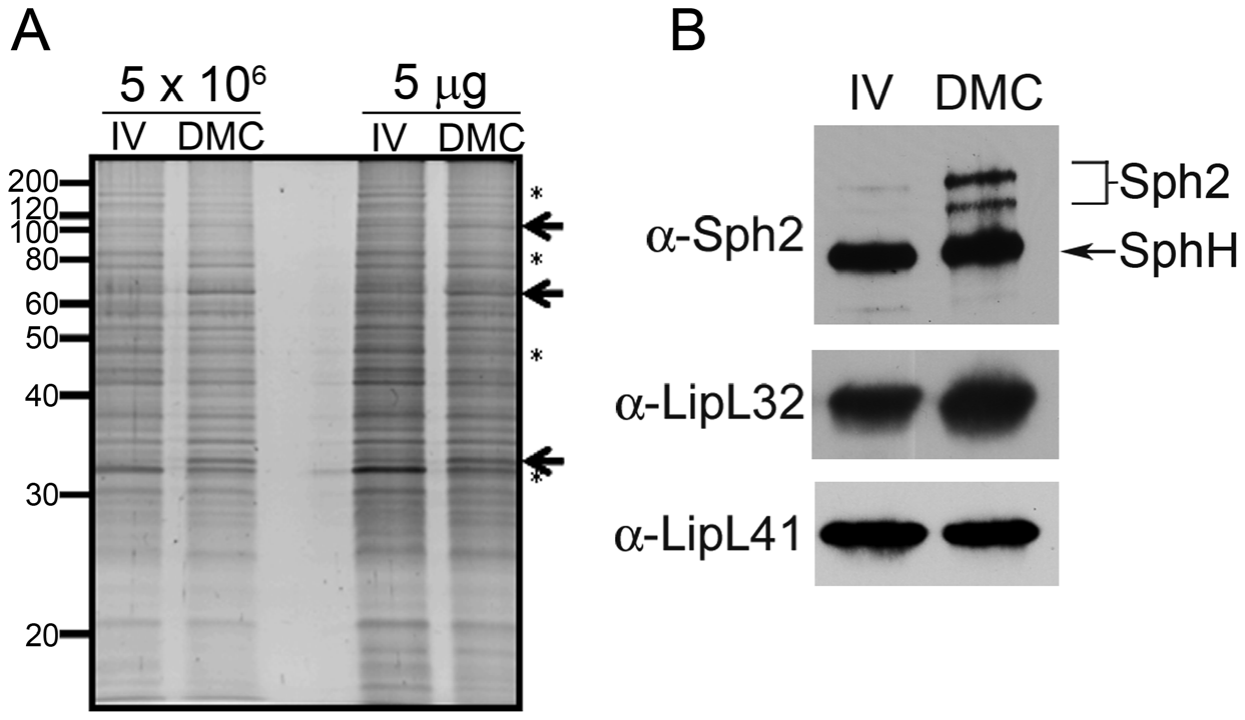
Virulent leptospires become mammalian host-adapted during growth within dialysis membrane chambers. Representative whole cell lysates of leptospires cultivated to late-logarithmic phase in EMJH medium at 30°C *in vitro* (IV) and within dialysis membrane chambers (DMC) implanted into the peritoneal cavities of female Sprague-Dawley rats. (A) Lysates were loaded according to the numbers of leptospires (5×10^6^ per lane) or total protein (5 µg per lane) and stained with SYPRO Ruby gel stain. Arrows and asterisks are used to highlight examples of polypeptides whose expression appears to be increased or decreased, respectively, within DMCs compared to *in vitro*. Molecular mass markers are indicated on the left. (B) Immunoblot analyses using rabbit polyclonal antisera directed against Sph2 [34], LipL32 [38] and LipL41 [39]. An arrow is used to indicate a band of the predicted molecular mass for SphH, a second, closely-related sphingomyelinase in *L. interrogans* recognized by antiserum directed against Sph2 [34], [37].

Our extensive experience with cultivation of Lyme disease spirochetes in DMCs implanted into rats [23], [24], [27], [32], a natural reservoir for *L. interrogans*, led us to ask whether the DMC model could be used to generate mammalian host-adapted *Leptospira*. In preliminary experiments, we determined that virulent low-passage *L. interrogans* sv. Copenhageni strain Fiocruz L1-130, diluted to low density (1×10^4^ leptospires/ml) in EMJH medium, undergoes exponential replication within DMCs, reaching a maximal density of ∼7×10^7^ leptospires/ml within 8 days post-implantation (data not shown). Importantly, leptospires recovered from DMCs explanted daily between 8 and 12 days post-implantation were vigorously motile by dark-field microscopy. The polypeptide profiles of leptospires in DMCs explanted between 9 and 12 days were highly similar (data not shown). On the basis of these studies, we chose 10 days as our standard period for intraperitoneal implantation. As shown in Figure 1A, under these conditions, we noted numerous polypeptides whose expression was either increased or decreased in response to mammalian host-derived signals compared to *in vitro*-grown bacteria. The polypeptide differences between *in vitro*- and DMC-cultivated organisms were even more apparent by two-dimensional SDS-PAGE (Figure S1). While a comprehensive quantitative analysis of these differentially-expressed polypeptides is necessary to identify the corresponding leptospiral proteins, these data support our contention that virulent leptospires substantially alter their proteome in response to mammalian host-specific signals.

With *B. burgdorferi*, successful mammalian host-adaptation within DMCs is determined by the reciprocal expression of the outer surface lipoprotein (Osp) A and OspC lipoproteins, which are OFF and ON, respectively, within the mammal [23]. However, no expression profile associated with host-adapted *L. interrogans* has been reported and only a handful of leptospiral genes/proteins have been shown to be reproducibly upregulated during mammalian infection. Among these is Sph2, one of four sphingomyelinase-like proteins encoded by *L. interrogans* sv. Copenhageni [12]. Although most strains of *L. interrogans* encodes at least 3 distinct sphingomyelinase-like proteins (Sph1, Sph2 and Sph3), only Sph2 is thought to be a “true” (*i.e.*, enzymatically active) sphingomyelinase [33]. Expression of Sph2 is upregulated *in vitro* in response to serum [21] and/or increased osmolarity [34] and during mammalian infection [35]. On the other hand, SphH, a closely-related pore-forming protein without sphingomyelinase activity [33], [36], is expressed constitutively *in vitro* [34], [37] and by leptospires colonizing the renal tubules of infected hamsters [37]. Consistent with these previous studies, the level of Sph2 was substantially higher in DMC-cultivated leptospires compared to *in vitro*-grown organisms, whereas SphH was expressed at similar levels under both conditions (Figure 1B). Immunoblots using antisera against LipL32 and LipL41, two leptospiral lipoproteins expressed constitutively *in vitro* and during mammalian infection [38], [39], [40], were performed as loading controls (Figure 1B). We considered these data as strong indication that DMC-cultivated leptospires are in a mammalian host-adapted state.

### RNA-Seq analysis of *Leptospira* cultivated *in vitro* and within DMCs

**Figure 2:**
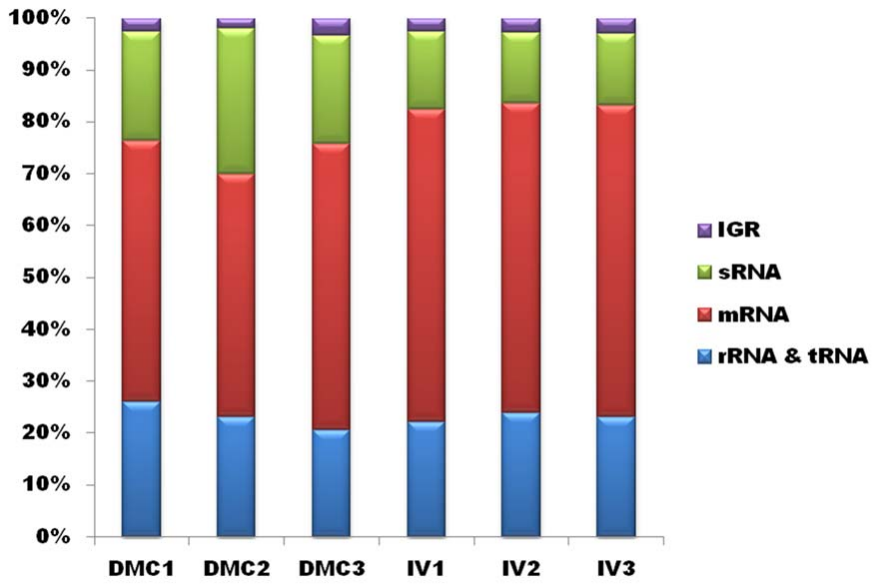
Mapping of RNA-Seq reads. Percentage of uniquely mapping reads from each biological replicate of leptospires cultivated in DMCs or under standard *in vitro* growth conditions (30°C in EMJH).

Having established the feasibility of using DMCs to generate mammalian host-adapted *L. interrogans*, we compared the transcriptional profiles of DMC- and *in vitro*-cultivated leptospires by RNA-Seq. To ensure that our data would be robust and reproducible, we generated Illumina TruSeq libraries from three biologically-independent samples for each growth condition. The sequence statistics and numbers of mapped reads for each biological replicate are summarized in Table 1 and displayed graphically in Figure 2. The total number of reads ranged from ∼8–14 million per library, of which 79–94% of reads mapped to the *L. interrogans* sv. Copenhageni Fiocruz L1-130 reference genome [11], [12]; only those reads that mapped to a single location on either Chromosome 1 or 2 were used to assess gene expression. The majority (43–55%) of unique sequence reads mapped to protein-coding mRNAs annotated on Chromosome 1, while ∼3–5% mapped to predicted ORFs on Chromosome 2; this 12∶1 ratio is consistent with the relative coding capacities of the two chromosomes [11], [12]. As discussed below, a considerable number of reads (13–20%) in both chromosomes mapped to non-coding regions that represent candidate small regulatory RNAs (sRNAs) (Table 1).

**Table 1:** Summary of RNA-Seq mapping data.

Library	Total # of Reads	Total # of mapped reads^1^	Uniquely mapped Reads^2^	Chromosome 1^3^				Chromosome 2^3^		
				mRNA	ncRNA	rRNA & tRNA	Intergenic	mRNA	ncRNA	Intergenic
DMC_1	9,150,966	8,327,861 (91.01%)	598,674 (6.54%)	279,333 (46.66%)	125,095 (20.90%)	155,452 (25.97%)	13,828 (2.31%)	22,797 (3.81%)	374 (0.06%)	1,795 (0.30%)
DMC_2	7,902,948	6,543,759 (82.80%)	574,766 (7.27%)	247,398 (43.04%)	161,761 (28.14%)	133,152 (23.17%)	9,997 (1.74%)	20,822 (3.62%)	382 (0.07%)	1,254 (0.22%)
DMC_3	14,089,133	11,125,795 (78.97%)	1,151,053 (8.17%)	589,345 (51.20%)	240,121 (20.86%)	235,839 (20.49%)	33,601 (2.92%)	46,718 (4.06%)	1,136 (0.10%)	4,293 (0.37%)
30°C_1	9,562,316	8,997,777 (94.10%)	870,275 (9.10%)	482,828 (55.48%)	129,770 (14.91%)	192,490 (22.12%)	20,249 (2.33%)	41,187 (4.73%)	770 (0.09%)	2,981 (0.34%)
30°C_2	11,642,652	10,702,058 (91.92%)	842,265 (7.23%)	456,665 (54.22%)	113,539 (13.48%)	201,691 (23.95%)	20,245 (2.40%)	45,486 (5.40%)	918 (0.11%)	3,721 (0.44%)
30°C_3	10,175,557	9,462,697 (92.99%)	805,288 (7.91%)	441,078 (54.77%)	109,817 (13.46%)	186,689 (23.18%)	21,165 (2.63%)	42,184 (5.24%)	809 (0.10%)	3,546 (0.44%)

1 Total number and percentage (in parenthesis) of reads that mapped to the reference genome with 100% accuracy.

2 Total number and percentage (in parenthesis) of reads that mapped to a single location within the reference genome [11], [12].

3 Based on the total of uniquely mapped reads for the corresponding sample.

### RNA-Seq provides comprehensive coverage of the leptospiral transcriptome under *in vivo* and *in vitro* growth conditions

The genome of *L. interrogans* sv. Copenhageni Fiocruz L1-130 harbors 3728 protein-encoding genes [11], [12]. The vast majority (∼94%) of these (3489 and 3499 in DMC- and *in vitro*-cultivated leptospires, respectively), were represented in our RNA-Seq data by a mean expression value of ≥1 (Table S2). We observed average mean expression values of 67.2 and 60.5 per gene in DMC- and *in vitro*-cultivated organisms, respectively (data not shown).

By comparative genomics, Picardeau *et al.* [14] identified 2052 “core” protein-coding genes that are shared between pathogenic (*L. interrogans* and *L. borgpetersenii*) and saprophytic (*L. biflexa*) *Leptospira* species. Not surprisingly, many of these core gene products are associated with housekeeping functions, such as motility, energetics and intermediary metabolism, DNA and RNA metabolism, and cell division [14]. Analysis of the protein-coding sequences for the 100 most highly-expressed genes (*i.e.*, Top 100) in DMC-cultivated leptospires revealed that 66 are conserved (*i.e.*, ≥40% amino acid identity over ≥80% of the coding region) between pathogenic and saprophytic *Leptospira* spp. and, therefore, part of the core group (Table S3); of note, the percentage (66%) of core genes within our Top100 is similar to the overall percentage (55%) of core genes within the entire *L. interrogans* sv. Copenhageni genome [14]. Consistent with their proposed housekeeping functions, 62 (94%) of the 66 core genes within the Top 100 were expressed at similar levels *in vitro* and within DMCs (Table S3). Thirty-four of the Top 100 genes are pathogen-specific (*i.e.*, no orthologous gene identified in *L. biflexa*), two of which (*LIC10465/ligA* and *LIC12653*) are found only in *L. interrogans* (*i.e.*, absent in *L. borgpetersensii, L. licerasiae* and *L. santarosai*). Eight of the 34 pathogen-specific genes within the Top 100 were upregulated by *L. interrogans* sv. Copenhageni within DMCs (see Table S3 and below).

We also surveyed both DMC- and *in vitro*-derived datasets for genes associated with key metabolic pathways. One unusual metabolic feature of pathogenic leptospires, compared to other spirochetes, is that they are unable to utilize glucose despite encoding a seemingly complete glycolytic pathway, relying instead on β-oxidation of long-chain fatty acids as sources of both carbon and energy [11], [41]. By RNA-Seq, we detected uniquely mapped reads for all of the genes thought to be involved in glucose uptake and utilization (KEGG pathway lic00010), each of which was expressed at similar levels in DMCs and *in vitro* (Table S4). However, two genes, *LIC13358* and *LIC20119*, both encoding putative phosphoglucomutases, and *LIC12908*, encoding the only glucose transporter identified in *L. interrogans* [11], [12], [42], were expressed at extremely low levels, both in DMCs and *in vitro* (Table S2). These data support the findings of Zhang *et al.* [42], who proposed that the inability of pathogenic leptospires to utilize glucose stems from insufficient glucose uptake and/or catalysis rather than an incomplete glycolytic pathway. As one might predict, we detected significant numbers of sequence reads for genes involved in the uptake and β-oxidation of medium and long-chain fatty acids (KEGG pathway lic00071), the citric acid cycle (KEGG lic00020), generation of NAD/NADP (KEGG lic00760), and oxidative phosphorylation (KEGG lic00190). All of the individual genes involved in these energetic pathways were expressed at similar levels under both growth conditions (Table S4).

### Genes whose expression was significantly upregulated by leptospires in DMCs compared to *in vitro*-grown bacteria

**Figure 3:**
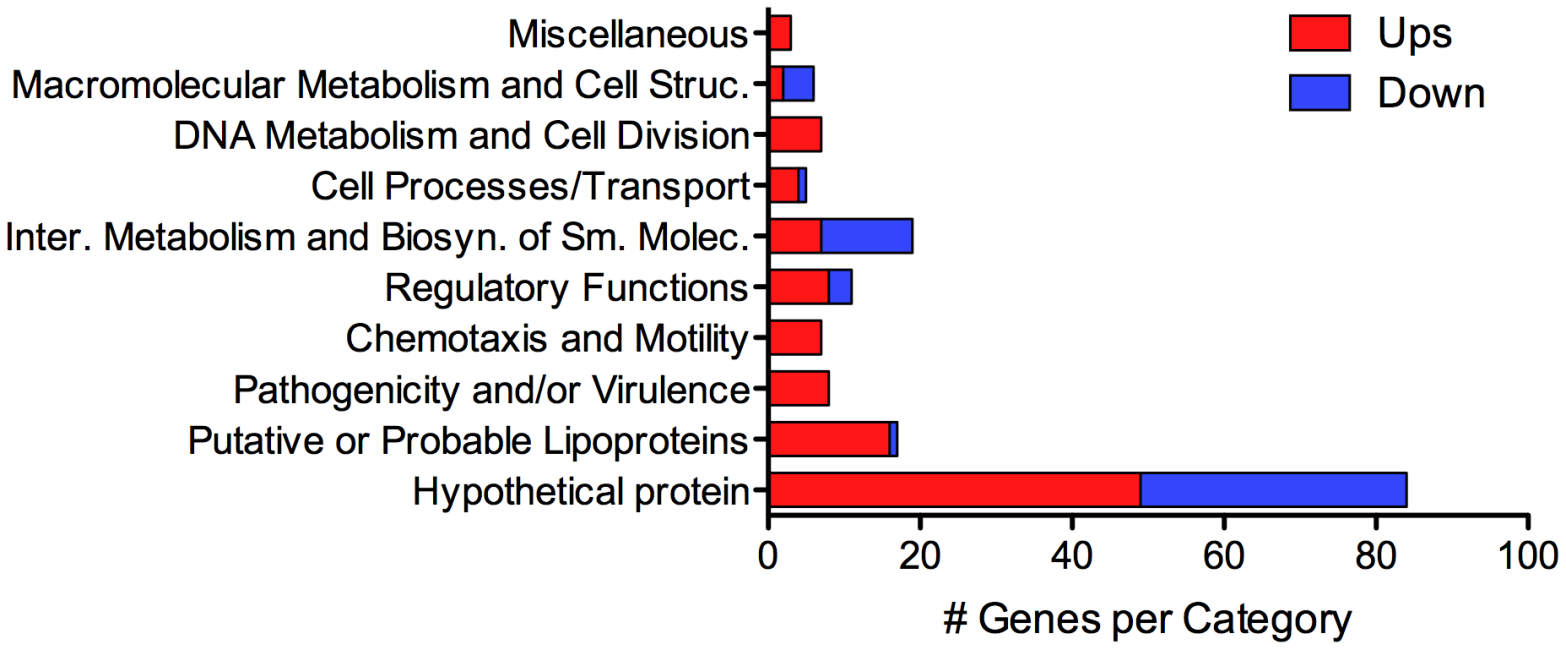
Conservation of *L. interrogans* sv. Copenhageni Fiocruz L1-130 differentially-expressed genes among virulent and saprophytic *Leptospira* spp. Protein sequence similarities were determined using GLSEARCH (v. 34.05). Genomes used for analysis: *L. interrogans* sv. Lai strain 56601, *L. borgpetersenii* sv. Hardjo strain L550, *L. santarosai* sv. Shermani strain LT821; *L. licerasiae* sv. Varillal strain VAR010; and *L. biflexa* sv. Patoc strain Patoc1 Ames, respectively. The color coding used in the heat map is as follows: blue, 95–100% identity; green, 90–94% identity; orange, 85–89%; and yellow, 80–84%.

**Figure 4:**
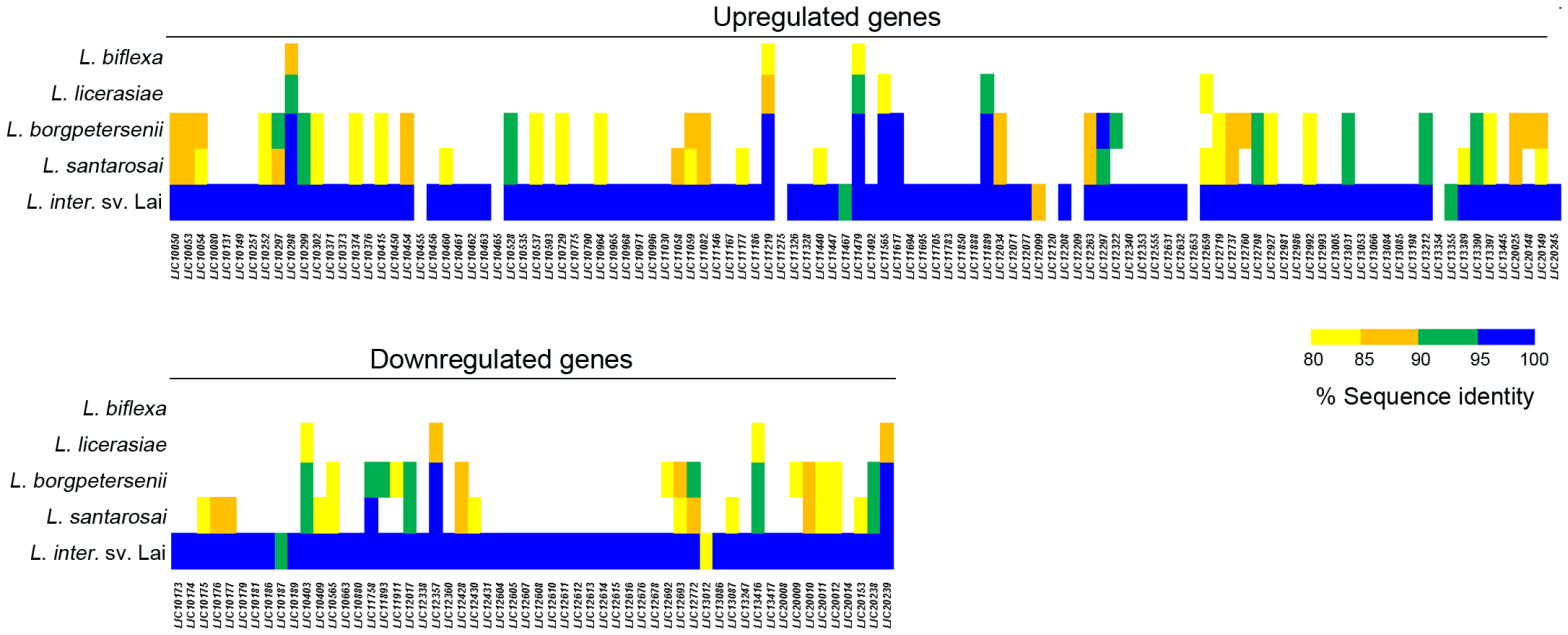
Functional categories of genes differentially-expressed by *L. interrogans* sv Copenhageni strain Fiocruz L1-130 within DMCs. Functional categories are based on those of [11], [12] and the *Leptospira interrogans* sv. Copenhageni Genome Project database (http://aeg.lbi.ic.unicamp.br/world/lic/). The number of upregulated (Ups) and downregulated (Down) genes within each category are indicated in red and blue, respectively.

Using DESeq [43], we identified 166 genes whose expression was either positively- or negatively-regulated by ≥2-fold (adjusted p-value≤0.05) within the mammal (Tables 2 and 3). Although some variance was observed between biological replicates (Table S2), a heat map representing the expression data for all 166 differentially-expressed genes confirmed that each biological replicate clustered with its respective sample source (Figure S2). Of the 110 genes upregulated by *L. interrogans* within DMCs, 106 are on Chromosome 1 while only 4 are on Chromosome 2 (Table 2). All but 3 of the upregulated genes appear to be pathogen-specific (*i.e.*, a paralogous gene/protein could not be identified in *L. biflexa*; 54 of these are unique to *L. interrogans* and an additional 7 are unique to serovar Copenhageni (Figure 3). Almost half (49/110) of the genes upregulated in DMCs encode hypothetical proteins (Figure 4 and Table 3), which is consistent with the overall percentage (40%) of hypothetical genes annotated within *L. interrogans* [6], [11]. Based on searches performed using the Conserved Domain Database [44], [45], none of the hypothetical proteins encoded by these genes contained readily identifiable functional domains (data not shown). However, one gene (*LIC12986*) recently was shown to be required for leptospires to survive within hamsters and to colonize the renal tubules of mice [46].

**Table 2:** *L. interrogans* sv. Copenhageni genes upregulated in DMCs compared to *in vitro*.

Gene ID^1^		Product^1^	Fold (DMC vs IV)	P value (adjusted)
***Hypothetical Proteins***				
*LIC10822*		Hypothetical protein	ND @ 30°C	4.00E-02
*LIC12077*		Hypothetical protein	22.78	1.19E-18
*LIC13005*		Hypothetical protein	21.12	2.75E-04
*LIC13445*		Hypothetical protein	17.46	6.13E-06
*LIC11059*		Hypothetical protein	16.76	2.65E-13
*LIC10456*		Hypothetical protein	10.59	4.12E-02
*LIC10965*		Hypothetical protein	9.73	8.94E-08
*LIC12340*		Hypothetical protein	7.14	3.40E-10
*LIC13390*		Hypothetical protein	6.60	2.74E-04
*LIC10455*		Hypothetical protein	6.54	1.04E-02
*LIC12120*		Hypothetical protein	6.20	1.93E-02
*LIC10376*		Hypothetical protein	6.14	2.07E-02
*LIC11888*		Hypothetical protein	5.71	1.33E-08
*LIC10535*		Hypothetical protein	5.61	2.65E-02
*LIC12555*		Hypothetical protein	4.76	5.29E-04
*LIC10971*		Hypothetical protein	4.35	8.70E-03
*LIC10775*		Hypothetical protein	4.27	8.69E-07
*LIC10790*		Hypothetical protein	4.15	8.98E-03
*LIC11695*		Hypothetical protein	4.06	9.65E-03
*LIC11492*		Hypothetical protein	4.03	4.56E-02
*LIC12986*		Hypothetical protein	4.03	2.53E-02
*LIC10415*		Hypothetical protein	3.93	5.73E-06
*LIC13354*		Hypothetical protein	3.77	4.53E-03
*LIC12653*		Hypothetical protein	3.63	5.83E-03
*LIC12993*		Hypothetical protein	3.53	1.19E-03
*LIC10593*		Hypothetical protein	3.32	3.07E-03
*LIC11705*		Hypothetical protein	3.14	3.55E-02
*LIC10080*		Hypothetical protein	3.14	5.99E-03
*LIC10374*		Hypothetical protein	3.09	2.12E-02
*LIC10729*		Hypothetical protein	2.97	2.12E-02
*LIC10450*		Hypothetical protein	2.94	1.21E-02
*LIC10454*		Hypothetical protein	2.86	4.53E-02
*LIC11783*		Hypothetical protein	2.82	4.12E-02
*LIC10053*		Hypothetical protein	2.81	1.23E-02
*LIC10460*		Hypothetical protein	2.74	5.31E-03
*LIC13084*		Hypothetical protein	2.72	2.03E-02
*LIC12263*	*ompL37*	Hypothetical protein	2.67	8.81E-03
*LIC20245*		Hypothetical protein	2.66	4.04E-02
*LIC12719*		Hypothetical protein	2.66	2.24E-02
*LIC11275*		Hypothetical protein	2.66	3.36E-02
*LIC13212*		Hypothetical protein	2.61	3.75E-02
*LIC12353*		Hypothetical protein	2.53	4.28E-02
*LIC11565*		Hypothetical protein	2.52	2.11E-02
*LIC10050*		Hypothetical protein with OmpA-like domain	2.46	1.54E-02
*LIC11177*		Hypothetical protein	2.43	2.67E-02
*LIC11447*		Hypothetical protein	2.42	3.36E-02
*LIC10302*		Hypothetical protein	2.23	2.28E-02
*LIC12071*		Hypothetical protein	2.08	3.02E-02
***Putative or Probable Lipoproteins (unknown function)***				
*LIC12099*	*lipL53*	Lipoprotein	19.50	1.03E-26
*LIC11030*		Lipoprotein	11.18	1.78E-04
*LIC12209*		Lipoprotein	7.76	2.75E-15
*LIC11058*	*lemA*	Lipoprotein	6.27	8.69E-07
*LIC10373*		Lipoprotein	5.37	4.61E-11
*LIC13066*		Lipoprotein	5.34	3.09E-09
*LIC10462*		Lipoprotein^2^	5.08	3.17E-07
*LIC10371*		Lipoprotein	4.61	9.64E-08
*LIC10054*	*rlpA*	Lipoprotein	3.84	1.44E-05
*LIC13355*		Lipoprotein	3.72	1.25E-02
*LIC12208*		Lipoprotein	3.64	2.07E-04
*LIC10461*		Lipoprotein	3.54	1.84E-04
*LIC10968*		Lipoprotein^2^	3.44	2.81E-03
*LIC10463*		Lipoprotein	3.31	4.81E-04
*LIC11082*		Lipoprotein	3.15	2.03E-03
*LIC11167*		Lipoprotein	2.24	2.03E-02
***Pathogenicity and/or Virulence***				
*LIC12760*	*colA*	Collagenase precursor	49.03	2.63E-51
*LIC12631*	*sph2*	Hemolysin/sphingomyelinase-like protein, Sph2	13.92	5.13E-26
*LIC11219*	*ahpC*	Peroxiredoxin	5.96	7.29E-07
*LIC10465*	*ligA*	*Leptospira* Ig-like protein LigA^3^	4.57	2.03E-02
*LIC12632*	*sph1*	Hemolysin/sphingomyelinase-like protein, Sph1	4.02	1.52E-05
*LIC12927*		Cytochrome c peroxidase^2^	4.23	1.04E-05
*LIC13198*	*sph3*	Hemolysin/sphingomyelinase-like protein, Sph3	3.57	1.99E-02
*LIC12659*	*vapB*	Virulence-associated protein	2.90	2.66E-02
***Chemotaxis and Motility***				
*LIC10299*	*flgB*	Flagellar basal body rod protein FlgB	7.89	4.89E-16
*LIC11889*	*flaB*	Flagellin protein	3.95	1.50E-02
*LIC10298*	*flgC*	Flagellar basal body rod protein FlgC	3.91	2.12E-05
*LIC11328*	*flgJ*	Flagellum-specific muramidase	3.89	7.28E-03
*LIC10297*	*fliE*	Flagellar hook-basal body protein FliE	3.16	1.87E-03
*LIC11326*	*flgH*	Flagellar L-ring protein precursor	2.66	3.03E-02
*LIC11186*	*flbC*	Flagellar protein	2.37	3.72E-02
***Regulatory Functions***				
*LIC12798*		TetR family transcriptional regulator	5.64	3.65E-05
*LIC11146*		DeoR family transcriptional regulator	4.42	3.08E-03
*LIC12034*	*fur*	Fur family transcriptional regulator	3.83	7.84E-04
*LIC11440*		Histidine kinase response regulator hybrid protein	3.36	1.04E-03
*LIC10996*		EAL-type diguanylate phosphodiesterase	3.23	2.11E-03
*LIC11617*		ArsR family transcriptional regulator	3.11	7.39E-03
*LIC20025*		Cyclic nucleotide binding protein	2.41	4.73E-02
***Intermediary Metabolism and Biosynthesis of Small Molecules***				
*LIC13053*	*desA*	Fatty acid desaturase	6.72	2.98E-11
*LIC12981*		Glutathione S-transferase	4.00	2.59E-02
*LIC13397*	*phoD*	Alkaline phosphatase	3.21	6.13E-04
*LIC13085*	*coaE*	Dephospho-CoA kinase	3.20	1.79E-02
*LIC20148*	*hol*	Heme oxygenase	3.06	3.35E-03
*LIC12322*		Glutaconate CoA transferase-like protein	2.32	4.80E-02
*LIC13031*		Aminotransferase	2.25	1.46E-02
*LIC13465*		Gly tRNA	4.69	1.69E-02
***Cell Processes/Transport***				
*LIC11694*		TonB-dependent outer membrane receptor	14.85	2.26E-13
*LIC10964*	*phuR*	TonB-dependent outer membrane hemin receptor	3.46	2.23E-02
*LIC20149*		Multidrug-efflux transporter	2.82	4.22E-02
*LIC12992*	*sulP*	Sulfate permease	2.81	4.05E-03
***DNA Metabolism and Cell Division***				
*LIC11467*	*rcc1*	Regulator of chromosome condensation	7.80	1.92E-03
*LIC12737*		Site-specific modification DNA-methyltransferase	5.12	6.37E-11
*LIC12297*		DNA repair protein	4.55	3.80E-02
*LIC10131*	*mesJ*	Cell cycle protein	3.41	3.62E-04
*LIC10252*		Exonuclease	3.31	5.86E-03
*LIC13389*	*mutS*	DNA mismatch repair protein	2.56	3.80E-02
*LIC11479*	*xerD*	Integrase/recombinase protein	2.91	3.02E-03
***Macromolecular Metabolism and Cell Structure***				
*LIC10537*		OmpA-like peptidoglycan-associated periplasmic protein	3.79	1.00E-03
*LIC10528*	*pbpB*	Penicillin-binding protein 3	2.46	1.99E-02
***Miscellaneous***				
*LIC10149*	*frnE*	Polyketide synthase	4.89	1.08E-02
*LIC10251*		Rad50-like protein	3.22	4.05E-03
*LIC11850*	*rmsE*	16S ribosomal RNA methyltransferase RsmE	2.41	8.14E-03

1 Gene designations and protein product descriptions are based on those of [11], [12] and the *L. interrogans* sv. Copenhageni Genome Project database (http://aeg.lbi.ic.unicamp.br/world/lic/), except where indicated.

2 nnotation based on Setubal *et al.* [47].

3 Revised annotation based on bioinformatics.

**Table 3:** Leptospiral genes differentially-expressed within DMCs compared to *in vitro*.

Genes	No of genes in each category		
	Upregulated (%)^1^	Downregulated (%)^1^	Total (%)^2^
Known or predicted function	45 (41%)	20 (36%)	66 (40%)
Unknown or poorly characterized function^3^	65 (59%)	36 (64%)	101 (60%)
**Total**	110	56	166

1 Percentage of genes based on the total number of genes in upregulated or downregulated category.

2 Percentage of genes based on the total number of differentially-expressed genes.

3 Hypothetical proteins and uncharacterized lipoproteins.

Of the remaining upregulated genes, 16 encode putative lipoproteins of unknown function [47] (Figure 4 and Table 3). Surface-exposed spirochetal lipoproteins have been implicated in a wide range of pathogenesis-related functions, including adherence to extracellular matrix components and nutrient acquisition [48]. However, because the mechanism(s) responsible for sorting individual spirochetal lipoproteins remain poorly understood, it is not possible to predict based on amino acid sequence alone which, if any, might function at the pathogen-host interface.

#### Virulence-associated genes

Eight DMC-upregulated genes encode proteins implicated in pathogenicity and/or virulence. *LIC12760*/*colA*, the most significantly upregulated gene (49-fold) in our studies, encodes a collagenase precursor. Degradation of host tissues by this enzyme is thought to promote bacterial colonization and/or dissemination as well as provide an additional source of nutrients (*e.g.*, amino acids) [49]. *LIC12631*, *LIC12632* and *LIC13198*, respectively, encode Sph2, Sph1 and Sph3. Lysis of host erythrocytes by Sph2 may enhance acquisition of fatty acids and heme/iron from the host. Narayanavari *et al.* [33] also raised the possibility that the non-catalytic Sphs (Sph1 and Sph3) function as adhesins *via* their interaction with host sphingomyelin. *LIC10465* encodes leptospiral immunoglobulin-like (Lig) protein A; this multifunctional, outer membrane-associated lipoprotein has been shown to promote binding to host molecules, including fibronectin, fibrinogen and extracellular matrix [50], [51], [52]. Moreover, antibodies against LigA are protective in a hamster model of acute infection [53]. *LIC12659/vapB* encodes a putative virulence-associated protein with similarity to the AbrB-like family of transcriptional regulators [54]; ArbB-like transcription factors, also referred to as transition state regulator proteins, have been identified in diverse bacteria but only orthologs from *Bacillus* have been characterized with respect to function and DNA-binding capabilities [55], [56]. *LIC11219* and *LIC12927*, encoding a peroxiredoxin (AhpC) and cytochrome c peroxidase, respectively, are discussed below.

#### Motility-related genes

**Figure 5:**
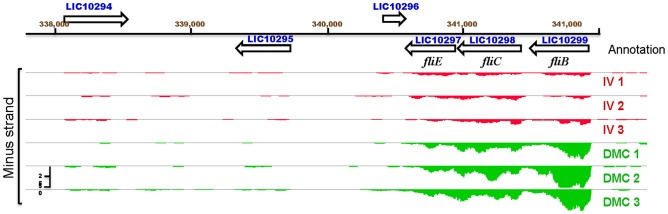
IGB viewer of normalized gene expression data for the flagellar genes *fliE*, *flgB* and *flgC*. Visualization of normalized mapped reads for minus (-) strand of an operon encoding genes *fliE*, *flgB* and *flgC* of the flagellar proximal rod shows increased expression by leptospires cultivated in dialysis membrane chambers (DMC, green) compared to those cultivated *in vitro* (IV, red). Annotated genes on Chromosome 1 are in blue. The vertical “read count” scale is 0–50.

Consistent with the highly invasive nature of leptospiral infection, seven motility-related genes were upregulated within DMCs (Table 2), including three (*LIC10299*/*flgB*, *LIC10298/flgC* and *LIC10297/fliE*) involved in flagellar basal body formation (Figure 5). The *L. interrogans* genome contains five copies of *flaB* (*LIC11889*, *LIC11890*, *LIC11531*, *LIC11532* and *LIC12947*), which encode the flagellar core subunit flagellin. Of these, only *LIC11889* was differentially-expressed within DMCs. Interestingly, based on the number of uniquely mapped reads determined by DESeq, *LIC12947* was expressed at substantially lower levels than the other four *flaB* paralogs under both growth conditions (Table S2), suggesting that this gene product may not contribute significantly to the formation of flagella *in vitro* or *in vivo*.

#### Uptake and utilization of iron/heme

Unlike *B. burgdorferi* [57], *L. interrogans* requires iron for growth *in vitro* and, presumably, within the mammalian host. In EMJH medium, leptospires obtain iron from Fe(II) sulphate, while organisms in the mammal acquire iron from heme and/or heme-containing proteins [58]. Heme (free or complexed with hemoglobin) is appropriated from the host by high-affinity TonB-dependent outer membrane receptor (TB-DR) proteins. Based on bioinformatic analysis, *L. interrogans* encodes at least 13 putative TB-DRs [58], however, only two (*LIC10964* and *LIC11694*) were upregulated within DMCs (Table 3 and Table S4). While most often associated with iron uptake, TB-DRs also may bind vitamin B_12_, a nutrient essential for leptospires *in vitro* and, presumably, *in vivo* [59]. Only one TB-DR in *L. interrogans* sv. Copenhageni (*LIC12374*/*btuB*) is annotated as being specific for vitamin B_12_, and the gene encoding this transporter component was not differentially-expressed in DMCs compared to *in vitro* (Table S4). Transport of heme and/or iron across the outer membrane requires energy produced by an inner membrane complex of the energy transduction protein TonB and two accessory proteins, ExbB and ExbD [59]. *L. interrogans* encodes at least two TonB-ExbB-ExbD complexes, arranged in separate operons, one on each chromosome. Interestingly, the transporter on Chromosome 2 (*LIC20216-20218*) was expressed at much higher levels (>14-fold) than its counterpart on Chromosome 1 (*LIC10889-10892*) under both growth conditions. Neither operon, however, was differentially-expressed in DMCs.

Consistent with an increased requirement for iron and/or heme *in vivo*, we detected increased expression (3.27-fold) of heme oxygenase (*LIC20148/hol*) [60] within DMCs. Once released, iron would be stored in the cytoplasm by bacterioferritin (*LIC11310*) and/or ferredoxin (*LIC13258* and *LIC13209*) gene products, all of which were well expressed by leptospires *in vitro* and in DMCS (Table S5).

When in excess, iron can lead to toxicity *via* the production of reactive oxygen species (ROS). As such, bacterial genes associated with iron homeostasis often are regulated by the ferric uptake regulator protein Fur, a global iron-responsive transcriptional repressor [61]. *L. interrogans* encodes at least four putative Fur paralogs (*LIC11006*, *LIC11158*, *LIC12034* and *LIC20147*). We used SLiMSearch [62] to survey the *L. interrogans* genome for “fur boxes” ([GC]AT[AT]AT[GC]AT[AT]AT[GC]AT[AT]AT[GC]) [61], and were unable to identify any obvious Fur-regulated genes (data not shown). Fur proteins, including those encoded by *Leptospira* spp. [58], share significant sequence similarity with orthologs for Zur, a zinc uptake regulator, and Per, an oxidative stress response regulator [63]. Based on bioinformatics and/or experimental evidence, two of Furs identified in *L. interrogans* sv. Copenhageni (*LIC12034* and *LIC20147*) appear to encode Per orthologs [22], [58]. One of these (*LIC12034*) was upregulated in DMCs (Table 2), suggesting that leptospires within DMCs are under some degree of oxidative stress.

#### Oxidative and thermal stress-related genes

Leptospires must cope with numerous stressors within the host, most notably, oxidative stress. Incomplete reduction of oxygen by iron-containing cytochromes is one potential source of endogenous ROS [64]. Leptospires likely encounter exogenously-derived ROS within the proximal renal tubules, a highly oxygenated tissue niche. Not surprisingly, *L. interrogans* encodes a more diverse repertoire of antioxidant proteins than either *Treponema pallidum* or *B. burgdorferi* (Table S5). Although *L. interrogans* encodes a functional catalase [65], it lacks superoxide dismutase (the enzyme typically associated with detoxification of O_2_^•−^) and the regulatory proteins OxyR and SoxR. Interestingly, only two oxidative stress-associated genes (*LIC12927* and *LIC11219*) were upregulated within DMCs (Table 2). The former encodes a cytochrome c peroxidase while the latter encodes an AhpC-type peroxiredoxin. In *E. coli*, AhpC scavenges basal levels of endogenous peroxide generated as a metabolic by-product [66]. Increased expression of AhpC within DMCs is consistent with increased uptake of exogenously-derived heme (see above) and increased potential for Fenton chemistry within the cytoplasm. Like *T. pallidum* [67], *L. interrogans* does not encode an AhpF, the usual reducing partner for AhpC, and most likely uses thioredoxin/thioredoxin reductase and/or glutaredoxin for this purpose, all of which were well expressed by DMC-cultivated leptospires (Table S5).

In addition to oxidative stress, increased temperature within the host might induce a stress response by leptospires *in vivo* [18], [19]. However, consistent with previous reports [19], [68], [69], none of the classical heat shock response genes encoded by *L. interrogans* were upregulated within DMCs, compared to *in vitro* growth at 30°C (Table S4).

#### Regulators of transcription

The leptospiral genome encodes >200 gene products with the potential to directly regulate transcription (Figure 3), including numerous two-component sensor histidine kinases (HKs) and/or response regulators (RRs), alternate sigma factors, sigma factor regulators, anti-sigma factor antagonists, and trans-acting factors [11], [12]. Only a few of these were upregulated within DMCs. One (*LIC11440*) encodes a hybrid sensor kinase/response regulator (HK/RR) protein; the sensor for this HK/RR contains a PAS-type sensor domain, which typically recognize small molecules, including heme [70]. Three additional genes (*LIC12798*, *LIC11146* and *LIC11617*) were DMC-upregulated and encode putative transcriptional regulators belonging to the TetR [71], DeoR [72], and ArsC [73] families of repressor proteins.

### Genes whose expression was significantly downregulated in DMCs compared to *in vitro*

By RNA-Seq, we identified 56 genes (47 on Chromosome 1 and 9 on Chromosome 2) that were downregulated in DMCs (Tables 3 and 4). All of the downregulated genes are pathogen-specific (*i.e.*, not found in *L. biflexa*); almost half (26/56) are unique to *L. interrogans* (*i.e.*, not in *L. borgpetersenii*, *L. santarosai* or *L. licerasiae*) (Figure 3). As with the upregulated gene subset, more than half (35/56) of the DMC-downregulated genes encode hypothetical proteins (Figure 4 and Table 3); of note, almost half (43%) of these appear to be transcribed in two polycistronic operons (*LIC10173-10177* and *LIC12604-12616*). Interestingly, all of the genes within these two putative operons are pathogen-specific. Only one lipoprotein (*LIC20153*) was expressed at lower levels in DMCs (compared to 16 upregulated).

**Table 4:** *L. interrogans* sv. Copenhageni genes downregulated in DMCs compared to *in vitro*.

Gene ID^1^		Product^1^	Fold (DMC vs IV)	P value (adjusted)
***Hypothetical Proteins***				
*LIC10173*		Hypothetical protein	−31.35	2.28E-09
*LIC10174*		Hypothetical protein	−29.41	4.12E-02
*LIC10176*		Hypothetical protein	−16.09	1.52E-05
*LIC10175*		Hypothetical protein	−12.71	4.24E-15
*LIC12611*		Hypothetical protein	−11.09	2.02E-11
*LIC12616*		Hypothetical protein	−9.22	3.54E-03
*LIC12615*		Hypothetical protein	−8.46	2.01E-12
*LIC12614*		Hypothetical protein	−8.13	1.59E-05
*LIC12610*		Hypothetical protein	−6.99	2.07E-06
*LIC10179*		Hypothetical protein	−6.81	5.27E-03
*LIC13417*		Hypothetical protein	−6.59	2.38E-08
*LIC11893*		Hypothetical protein	−6.21	4.89E-04
*LIC12612*		Hypothetical protein	−6.19	2.50E-03
*LIC13247*		Hypothetical protein	−6.01	1.79E-02
*LIC10177*		Hypothetical protein	−6.01	4.11E-06
*LIC12608*		Hypothetical protein	−5.54	8.50E-04
*LIC12613*		Hypothetical protein	−5.46	1.09E-05
*LIC13416*		Hypothetical protein	−5.04	5.73E-06
*LIC12604*		Hypothetical protein	−4.94	2.02E-03
*LIC10189*		Hypothetical protein	−4.83	9.23E-03
*LIC12692*		Hypothetical protein	−4.76	2.40E-05
*LIC12338*		Hypothetical protein	−4.75	3.31E-02
*LIC10880*		Hypothetical protein	−4.49	3.89E-02
*LIC10186*		Hypothetical protein	−4.27	1.17E-02
*LIC12430*		Hypothetical protein	−3.51	1.99E-02
*LIC10187*		Hypothetical protein	−3.38	2.03E-02
*LIC12693*		Hypothetical protein	−3.36	2.65E-03
*LIC13012*		Hypothetical protein	−3.34	8.21E-03
*LIC12678*		Hypothetical protein	−3.30	4.75E-02
*LIC12607*		Hypothetical protein	−3.21	3.49E-02
*LIC12605*		Hypothetical protein	−3.14	8.38E-03
*LIC10181*		Hypothetical protein	−3.13	4.52E-02
*LIC12676*		Hypothetical protein	−2.87	1.49E-02
*LIC13086*		Hypothetical protein^2^	−2.77	2.11E-03
*LIC10663*		Hypothetical protein	−2.65	1.69E-02
***Putative or Probable Lipoproteins (unknown function)***				
*LIC20153*		Lipoprotein^2^	−2.90	2.27E-02
***Regulatory Functions***				
*LIC13087*		Histidine kinase sensor protein	−4.55	2.15E-02
*LIC12431*		TetR family transcriptional regulator	−2.87	1.49E-02
*LIC20012*		Histidine kinase sensor protein	−2.52	4.03E-02
***Cellular Processes/Transport***				
*LIC12428*	*phnL*	ABC transporter ATP-binding protein	−7.96	2.03E-02
***Intermediary Metabolism and Biosynthesis of Small Molecules***				
*LIC20008*	*hemA*	Glutamyl-tRNA reductase	−6.31	2.78E-09
*LIC11758*		Acyl-CoA hydrolase	−5.56	2.23E-02
*LIC12772*	*proB*	Gamma-glutamyl kinase	−3.97	2.53E-02
*LIC20009*	*hemC*	Porphobilinogen deaminase	−3.74	1.82E-04
*LIC10409*	*leuA*	2-isopropylmalate synthase	−3.34	1.93E-03
*LIC20014*	*hemE*	Uroporphyrinogen decarboxylase	−3.33	3.41E-03
*LIC10565*	*hbd1*	Enoyl-CoA hydratase	−3.28	2.26E-02
*LIC20238*	*speH*	S-adenosylmethionine decarboxylase like protein	−3.28	1.19E-03
*LIC10403*	*ribH*	6,7-dimethyl-8-ribityllumazine synthase	−2.86	3.06E-02
*LIC20011*	*hemL*	Glutamate-1-semialdehyde aminotransferase	−2.44	1.49E-02
*LIC20239*	*speD*	S-adenosylmethionine decarboxylase proenzyme	−2.31	1.99E-02
*LIC20010*	*hemB*	Delta-aminolevulinic acid dehydratase	−2.29	3.57E-02
***Macromolecular Metabolism and Cell Structure***				
*LIC12017*	*clpB*	ATP-dependent protease	−4.71	1.11E-02
*LIC12360*	*pirin*	Pirin	−2.83	6.60E-03
*LIC12357*	*fusA*	Elongation factor EF-G	−2.71	3.53E-03
*LIC11911*		Glycosyltransferase	−2.65	4.04E-02

1 Gene designations and protein product descriptions are based on those of [11], [12] and the *L. interrogans* sv. Copenhageni Genome Project database (http://aeg.lbi.ic.unicamp.br/world/lic/) except where indicated.

2 Annotation based on Setubal *et al.* [47].

Five genes related to *de novo* heme biosynthesis (*LIC20008/hemA*, *LIC20009/hemCD*, *LIC20010*/*hemB*, *LIC20011/hemL* and *LIC20014/hemE*) [74] were DMC-downregulated these findings imply that leptospires can scavenge heme from the mammalian host. The heme biosynthetic operon also contains genes encoding a two component system (TCS). Signal transduction by the orthologous TCS in *L. biflexa* is required for regulation of heme biosynthesis [75]. Although both the histidine kinase (HK; *LIC20012*) and the response regulator (RR; *LIC20013*) were downregulated (2.50- and 2.22-fold; respectively) in DMCs, the fold-change for the RR was not significant (p = 0.097). Based on their tandem arrangement and similar expression profiles, these heme biosynthetic genes appear to be transcribed as a single operon. *LIC20017/hemG* and *LIC20018/hemH*, encoding enzymes responsible for the last two steps in heme biosynthesis, respectively, are located downstream of the larger biosynthetic operon; both of these genes appear to be transcribed as monocistronic messages at similar levels *in vitro* and in DMCs (Table 4 and data not shown).

### Identification of novel candidate small RNAs

**Figure 6:**
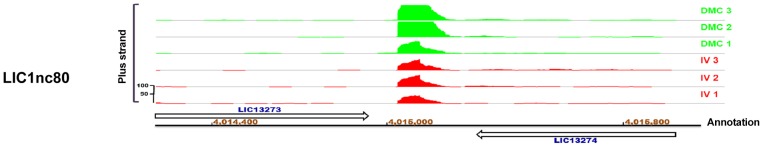
IGB viewer of candidate sRNA *LIC1nc80*. *LICnc80* was identified as an area of high transcriptional activity within an intergenic region of the genome of *L. interrogans* sv. Copenhageni Fiocruz L1-130. Expression data for leptospires cultivated in DMCs (green) compared to those cultivated *in vitro* (IV, red) are indicated on the plus strand of the genome. Annotated genes on the relevant chromosome and nucleotide co-ordinates are indicated. The vertical “read count” scale is 0–100.

One of the advantages of RNA-Seq is that it allows visualization of uniquely mapped reads within non-annotated regions of the genome. Using the IGB browser, we detected at least 11 regions that were transcriptionally-active but not protein coding; these non-coding RNA (ncRNA) transcripts are novel candidate small regulatory RNAs (sRNAs) within *L. interrogans* (Table 5 and Figure S3). Five of these are homologous to known sRNA families (tmRNA, RNaseP, PyrR binding site and two cobalamin sRNAs) (http://rfam.sanger.ac.uk/) [76], [77], [78], [79]. The expression of 8 of the 11 putative sRNAs was validated by reverse-transcriptase PCR in *L interrogans* sv. Copenhageni strain RJ16441 (Table 5) and all predicted sRNAs were highly conserved in the closely-related virulent serovar type strain Lai [80]. One of the predicted sRNAs, *LIC1nc80* (Figure 6), was significantly DMC-upregulated (4.39-fold) compared to *in vitro*-cultivated leptospires (Table S2). Further characterization of these candidate sRNAs (*i.e.*, by Northern blot) is required to understand their function(s) and relationships to the surrounding genes (*i.e.*, 5′ UTR verses bone fide sRNA).

**Table 5:** Candidate small non-coding RNAs identified by RNA-Seq.

Transcript^1^	Homology^2^	E-value	Chr	Genome Coordinates		sRNA size	Validated^3^
*LIC1nc10*	tmRNA	1.50E-62	1	175,606	175,960	355	+
*LIC1nc20*	PyrR binding site	1.50E-04	1	263,598	264,013	416	+
*LIC1nc30*	−		1	849,634	849,900	267	+
*LIC1nc50*	−		1	2,109,156	2,109,444	289	+
*LIC1nc55*	Cobalamin	3E-25	1	2,878,556	2,878,746	191	−
*LIC1nc60*	RNaseP	5.50E-31	1	3,031,445	3,031,846	402	+
*LIC1nc80*	−		1	4,015,037	4,015,237	201	−
*LIC2nc10*	Cobalamin	6.80E-19	2	159,019	159,243	225	+
*LIC2nc20*	−		2	242,735	243,092	358	+
*LIC2nc30*	−		2	246,062	246,477	416	+
*LIC2nc40*	−		2	348,946	349,168	223	−

1 Predicted sRNAs are annotated according to the genome of *L. interrogans* sv. Copenhageni (LIC) chromosome number followed by non-coding RNA designation as included in Supplementary Table S2.

2 Homology to known sRNA families is indicated as is the E-value when transcripts were searched against the Rfam database.

3 Expression was validated by reverse-transcriptase PCR in *L. interrogans* sv. Copenhageni strain RJ16441.

### Validation of RNA-Seq data by quantitative RT-PCR

**Figure 7:**
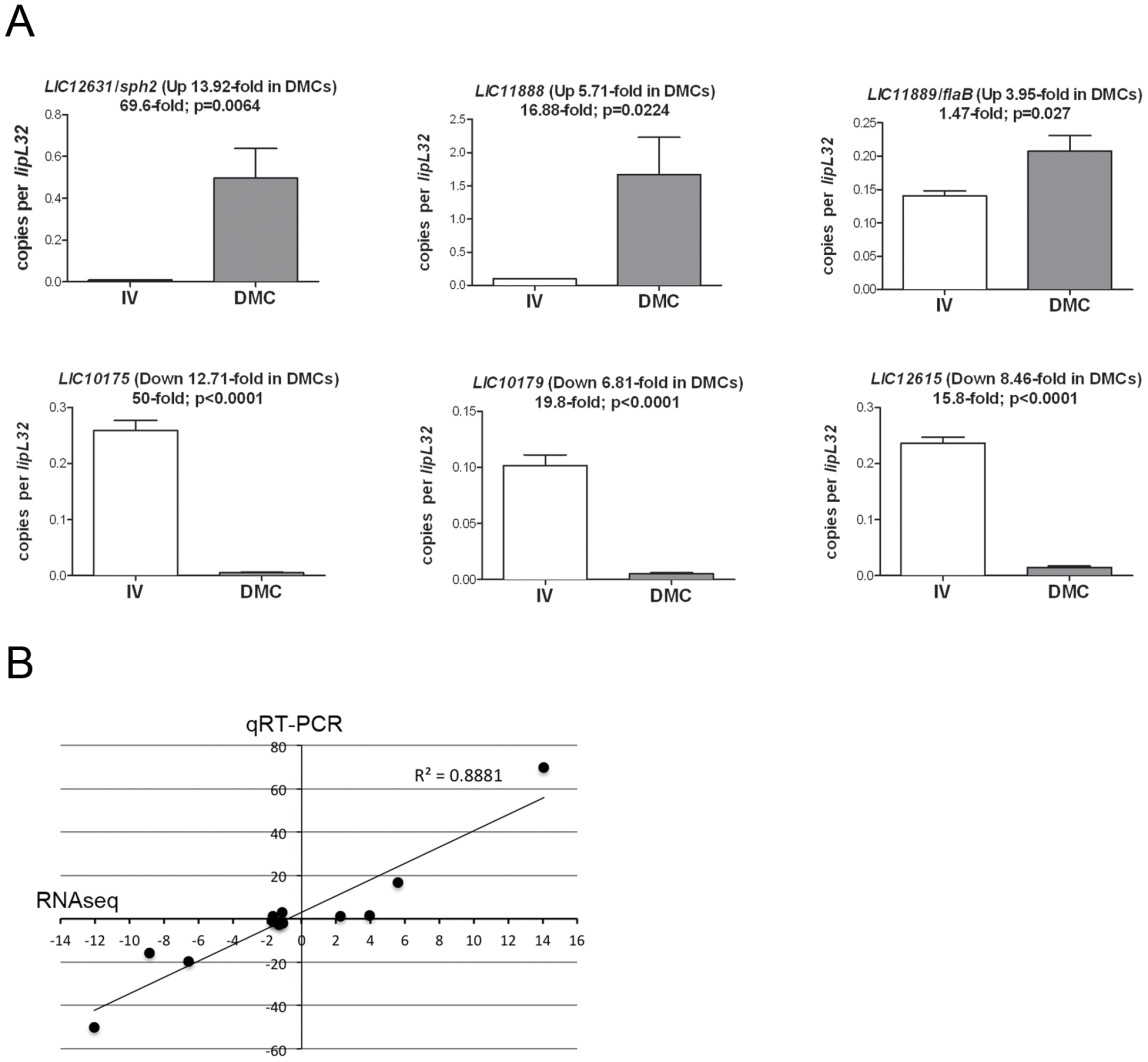
Validation of comparative RNA-Seq analysis. (A) qRT-pCR analysis of representative genes identified by RNA-Seq. Values represent the average transcript copy numbers for each gene normalized per *lipL32* transcript. Bars indicate the standard error of the mean (SEM). Results presented are mean values from at least 3 biologically-independent samples of leptospires for each growth condition. The fold-regulation for each gene determined by RNA-Seq is indicated in parentheses. The fold-regulation between *in vitro*- (IV) and DMC-cultivated leptospires determined by qRT-PCR are indicated. P values were calculated using an unpaired *t*-test. (B) Correlation coefficient (R^2^) between RNA-Seq and qRT-PCR data.

To validate our RNA-Seq data, we performed quantitative reverse transcription-PCR (qRT-PCR) on a panel of 14 genes that were, according to DESeq analysis, upregulated (*LIC12631*/*sph2*, *LIC11888* and *LIC11889/flaB*), downregulated (*LIC10175*, *LIC10179* and *LIC12615*), or unchanged (*LIC10191/loa22*, *LIC12966/lipL41*, *LIC13166/ompL36*, *LIC10787/flaA-2*, *LIC10068*, *LIC10421*, *LIC12339*, and *LIC20001*) in DMCs compared to *in vitro*.While there is some debate regarding the most appropriate leptospiral gene to use for normalization [21], [81], we selected *LIC11352/lipL32* based on studies demonstrating that its expression was relatively unchanged under a wide-range of growth conditions, including increased temperature, increased osmolarity, and/or exposure to serum [21], [38], . Representative results are shown in Figure 7A; data for the entire panel are presented in Figure S4. Overall, we saw strong agreement between our RNA-Seq and qRT-PCR datasets; the correlation coefficient (R^2^) between RNA-Seq and qRT-PCR data across the entire panel was 0.8881 (Figure 7B). We also used qRT-PCR to confirm the relative expression for two (*LIC1nc60*/RNase P and *LIC2nc10*/cobalamin) of the putative sRNAs (Figure S4); of these, only *LIC1nc60*/RNaseP was upregulated (2.65-fold; p = 0.0054) within DMCs.

## Discussion

The identification of genes/proteins that are differentially-expressed by microorganisms only during infection and/or within specific host niches often provides insight into the parasitic strategies of pathogens. During natural and experimental infection in rats, *L. interrogans* rapidly disseminate hematogenously to all tissues but are cleared by 7 days post-inoculation from all sites except the kidneys [7], [82]. The ability of leptospires to colonize and persist within renal tubules almost certainly involves unique virulent determinants [1]; however, the paucilbacillary nature of leptospiral infection, even within this preferred niche, hinders our ability to perform global gene expression studies on *L. interrogans* within host tissues. Prior studies, including several using microarray-based approaches [18], [19], [21], [22], [83], have manipulated *in vitro* growth conditions to simulate the environmental signals encountered by leptospires within the mammal. Based on extensive studies with *B. burgdorferi*, another pathogenic spirochete, we and others have demonstrated that *bone fide* mammalian host adaptation is a complex and dynamic process that cannot be fully reproduced *ex vivo* [23], [27], [32]. We therefore used a rat peritoneal dialysis membrane chamber (DMC) model to generate sufficient *L. interrogans* in a mammalian host-adapted state to perform global transcriptional studies. Cultivation of leptospires within DMCs, in conjunction with next generation sequencing, enabled us to define for the first time the transcriptome of *L. interrogans* within the mammalian host.

In order to transition from a free living to infectious state, leptospires must adjust their metabolism to utilize nutrients available within the mammalian host. Quite surprisingly, we found that the majority of genes implicated in central and intermediary metabolism were expressed by leptospires at similar levels in DMCs and *in vitro*. We interpret these data to suggest that EMJH, the medium commonly used to cultivate pathogenic and saprophytic leptospires *in vitro*, reflects the overall composition of nutrients available within mammalian host fairly well. Nonetheless, leptospires cultivated within DMCs differentially-regulated a handful of genes whose products are involved in metabolic and biosynthetic pathways, most notably, heme uptake and utilization (see below). Although increased temperature often is implicated as an important stimulus for host adaptation, we observed very little overlap (<10%) between the cohort of genes that were upregulated in DMCs and those previously identified as being temperature-regulated *in vitro* [18], [19], [21], [68]. Thus, differential gene regulation by leptospires within DMCs appears to be driven primarily by non-thermal mammalian host-specific stimuli. The relatively small pore size of the dialysis tubing used to cultivate leptospires within rat peritoneal cavities would exclude macromolecules and most serum proteins but allow for efficient exchange of nutrients (*i.e.*, glucose, ions, and free amino acids) present within serum. These are the same types of small molecules that leptospires likely encounter within proximal convoluted tubules, where the composition of the glomerular ultrafiltrate most closely resembles that of interstitial fluid [84]. Further experimentation is required to assess how closely DMC-cultivated leptospires resemble their counterparts within host tissues during acute and/or chronic infection. The DMC model does have some limitations. For instance, virulence genes associated with pulmonary haemorrhage may be expressed only within the context of lung tissue. Because bacteria within DMCs are prevented from interacting with host immune cells and immunoglobulin [85], this model does not enable us to identify genes that are differentially-regulated in response to specific pathogen-host interactions and/or immune evasion.

Although increased temperature often is implicated as an important stimulus for host adaptation, we observed very little overlap (<10%) between the cohort of genes that were upregulated in DMCs and those previously identified as being temperature-regulated *in vitro* [18], [19], [21], [68]. We observed a similarly limited overlap between our RNA-Seq data and genes found to be differentially regulated *in vitro* in response to exposure to serum [21] and low iron [22]. We observed a somewhat higher, but nonetheless small, degree of overlap (16%) between our RNA-seq dataset and genes identified by Matsunaga *et al.* [20] as being upregulated by physiologic osmolarity (EMJH supplemented with 120 mM NaCl); included in this overlap are *lipL53* (*LIC12099*), *sph2* (*LIC12631*), a putative CoA-transferase (*LIC12322*), *phoD* (*LIC13397*) and *hol* (LIC20148; see below). Thus, differential gene regulation by leptospires within DMCs appears to be driven by mammalian host-specific stimuli that are not readily reproduced *in vitro*.

The relatively small pore size of the dialysis tubing used to cultivate leptospires within rat peritoneal cavities would exclude macromolecules and most serum proteins but allows for efficient exchange of nutrients (*i.e.*, glucose, ions, and free amino acids) present within serum. These are the same types of small molecules that leptospires likely encounter within proximal convoluted tubules, where the composition of the glomerular ultrafiltrate most closely resembles that of interstitial fluid [84]. Further experimentation is required to assess how closely DMC-cultivated leptospires resemble their counterparts within host tissues during acute and/or chronic infection. The DMC model does have some limitations. For instance, virulence genes associated with pulmonary haemorrhage may be expressed only within the context of lung tissue. Because bacteria within DMCs are prevented from interacting with host immune cells and immunoglobulin [85], this model does not enable us to identify genes that are differentially-regulated in response to specific pathogen-host interactions and/or immune evasion.

To date, >20 named species of *Leptospira* have been identified based on molecular taxonomic analyses [86]. *Leptospira* spp. can be further divided into three major groups based on pathogenicity: pathogenic (9 species), intermediate virulence (5 species) and free-living saprophytes (6 species). The vast majority (69%) of genes upregulated by leptospires in response to mammalian host signals are found only in pathogenic and intermediate virulence species (*i.e.*, absent in *L. biflexa*), suggesting that their gene products may help promote infection and/or colonization within mammal. However, more than half (64/110) of these upregulated genes encode either hypothetical proteins or lipoproteins of unknown function without any obvious conserved/functional domains. While their functions remain to be determined, our finding that these protein-coding genes are differentially-regulated in response to mammalian host-specific signals make them attractive candidates for further experimentation in animals model and, in particular, their potential use as part of a mono- or multi-valent protein-based vaccine. Thirty-five of the 56 genes downregulated in DMCs encode hypothetical proteins. Interestingly, all but 7 of these are unique to pathogenic and intermediate virulence species, raising the possibility that these genes products, while not required for survival within the host, facilitate the transition from a free-living to infective state.

Heme is the major source of iron in *L. interrogans* and also serves as a cofactor for proteins essential for respiration (*i.e.*, cytochromes), biosynthesis of vitamin B_12_, and detoxification of reactive oxygen intermediates (*i.e.*, catalase). Unlike *B. burgdorferi* [87] and *T. pallidum* [88], *L. interrogans* possess a complete set of genes required for *de novo* heme biosynthesis as well as the uptake and utilization of exogenous heme [58], [74], [89]. By RNA-Seq, expression of 6 heme biosynthesis genes was significantly downregulated in DMCs compared to *in vitro*, while heme oxygenase (*LIC20148/hol*) and *phuR*, encoding a TonB-dependent heme receptor, were upregulated; these data support the notion that pathogenic leptospires preferentially use exogenously derived heme within the mammal. Of the four putative *fur* orthologs encodes by *L. interrogans*, only one (*LIC12034*) was upregulated in DMCs. Recently, Marcsisin *et al.* [46] demonstrated that inactivation of this gene had no effect on virulence in a hamster acute infection, implying that this Fur paralog is not responsible for downregulation of the heme operon within DMCs. Alternatively, downregulation of heme biosynthesis is not a prerequisite for survival *in vivo*. Because heme is highly toxic [90], there is relatively little, if any, free heme within plasma [91]. In the glomerulus, the molecular weight cut-off for ultrafiltration is ∼70 kDa [92]. Thus, while *L. interrogans* is able to use haemoglobin (64 kDa) as a source of heme *in vitro* [60], this micronutrient is likely present in only minute amounts within the proximal tubules. Smaller molecules (≤20 kDa), only the other hand, easily pass through the glomerulus into Bowmen's capsule; it is worth noting that this molecular weight cut-off is essentially equivalent to that of the dialysis tubing used for our DMCs (8 kDa MWCO). Instead, leptospires may be using myoglobin (16.7 kDa), which is present in human plasma at concentrations similar to that of haemoglobin [93]. Both hemoglobin and myoglobin, released by red blood cell turnover and muscle tissue damage, respectively, are filtered by the kidneys and would be available to leptospires within the renal tubules.

Small non-coding RNAs (sRNAs) are increasingly recognized as essential post-transcriptional gene expression regulators that enable bacteria to adjust their physiology in response to environmental cues [94]. Bacterial sRNAs range from 50 to 500 nucleotides and frequently are located within intergenic regions [95]. By diverse mechanisms, including changes in RNA conformation, interactions with DNA, other RNAs and proteins, sRNAs can modulate transcription, translation, mRNA stability and DNA maintenance or silencing [96], [97]. Five of the 11 candidate sRNAs identified as part of this study are conserved in bacteria and known to carry out specific housekeeping functions, including RNase P (*LIC1nc60*), responsible for processing of tRNAs and other RNAs, and tmRNA (*LIC1nc10*), which acts as both a transfer RNA (tRNA) and mRNA to tag incompletely-translated proteins for degradation and to release stalled proteins [76], [77]. We also identified two cobalamin riboswitches (*LIC1nc55* and *LIC2nc10*), which act as *cis*-regulatory elements in 5′ untranslated regions of vitamin B_12_-related genes; allosteric rearrangement of mRNA structure is mediated by ligand binding resulting in modulation of gene expression or translation of mRNA [78]. *LIC1nc55* lies upstream of *LIC121374/btuB*, which encodes a constitutively-expressed TonB-dependent outer membrane cobalamin receptor protein [98]. We also identified a candidate sRNA (*LIC2nc10*) upstream of *LIC20135*; although annotated as a ferredoxin, LIC20135 contains a domain conserved within sirohydrochlorinin cobalt chelatases, an important enzyme involved in biosynthesis of vitamin B_12_. Finally, *LIC1nc20* contains a conserved PyrR binding site; this RNA element is found upstream of genes involved in pyrimidine biosynthesis and transport in *Bacillus subtilis* [79]. In *L. interrogans*, this sRNA was found downstream of genes encoding hypothetical proteins. In addition to these known sRNAs, we identified six transcriptionally-active, non-coding regions that encode novel candidate regulatory sRNAs. *LIC1nc30*, *LIC1nc50*, *LIC2nc30* and *LIC2nc40* were all identified in the 5′ untranslated regions for *LIC14007*, *LIC10702*, *LIC20192* and *LIC20276*, respectively, all of which encode proteins of unknown function. The remaining two putative sRNAs (*LIC1nc80* and *LIC2nc20*) are located in the 3′ untranslated region of genes, which are known to be a repository of sRNAs in other bacterial species [99].

The *L. interrogans* genome encodes >200 proteins whose annotations suggest a role in transcriptional regulation (*i.e.*, sigma factors, anti-sigma factors and trans-acting factors), two-component signal transduction and the synthesis/degradation of cyclic nucleotides [11], [12]. By RNA-Seq, the vast majority of these putative regulatory proteins were expressed at similar levels *in vitro* and in DMCs; this finding is not unexpected given that these types of regulatory factors typically are activated at the protein level by endogenously- or exogenously-derived small molecules and environmental stimuli [100], [101], [102].

Recent advances in *Leptospira* molecular genetics, including the development of site-directed [103] and transposon-mediated [104], [105], [106] mutagenesis techniques, now make it possible to determine the contribution(s) of genes that are regulated within DMCs. We anticipate that this approach will identify proteins involved in environmental sensing, mammalian host adaptation and/or the expression of specific virulence determinants *in vivo*.

## Materials and Methods

### Ethics statement

All animal experimentation was conducted following the Guide for the Care and Use of Laboratory Animals (Eighth Edition) and in accordance with protocol (ACC# 100570-0116) reviewed and approved by the University of Connecticut Health Center Institutional Animal Care and Use Committee. The UCHC laboratory animal care program is accredited by the Association for Assessment and Accreditation of Laboratory Animal Care. The USDA Site ID: Customer Number 44, Certificate Number 16-R-0025, PHS Assurance Number A3471-01.

### Bacteria

Virulent low-passage *Leptospira interrogans* sv. Copenhageni strains Fiocruz L1-130, kindly provided by Dr. David Haake (UCLA), and RJ16441 were cultivated *in vitro* under standard conditions at 30°C in EMJH medium [107] supplemented with 1% rabbit serum (Pel-Freez Biologicals, Rogers, AR) with 100 µg/ml 5-fluorouracil. Cultures were passaged *in vitro* no more than 3 times before being used for experimentation.

### Cultivation of virulent *L. interrogans* within dialysis membrane chambers

To obtain *L. interrogans* in a mammalian host-adapted state, organisms were cultivated in dialysis membrane chambers (DMCs) as previously described [23], [24]. Briefly, DMCs were constructed using standard dialysis membrane tubing (Spectra-Por; 8000 MWCO). Prior to use, 8-inch strips of dialysis tubing were tied off at one end and then sterilized by boiling for 20 min in sterile water containing 5 mM EDTA, followed by two successive boiling washes in water alone. Dialysis bags were cooled to room temperature and then filled with ∼8–9 mls of EMJH medium (supplemented with 10% vaccine-grade bovine serum albumin to maintain osmotic pressure) containing 10^4^ organisms per ml. Once filled, the tubing was tied and excess membrane removed from both ends. For implantation, female Sprague-Dawley rats (150–174 g) were anesthetized by intramuscular injection of a mixture of ketamine (50 mg/kg), xylazine (5 mg/kg), and acepromazine (1 mg/kg). Using strict aseptic technique, a DMC was implanted into the peritoneal cavity of each rat. Analgesia (carprofen; 5 mg/kg) was administered on the day of surgery and once the following day. At designated time points (typically 9–10 days post-implantation), rats were euthanized by CO_2_ narcosis and DMCs recovered. The contents of each chamber were removed by gentle syringe aspiration with an 18G needle; the needle was removed prior to expelling the DMC dialysate into a sterile 15 ml conical bottom tube. Bacteria were enumerated by dark field microscopy immediately following explant using a Petroff-Hausser counting chamber (Hausser Scientific Co., Horsham, PA).

### Gel electrophoresis and immunoblotting

*In vitro*-cultivated *L. interrogans*, harvested at late-log phase (5×10^8^–1×10^9^ per ml) and leptospires explanted from DMCs were processed for one- and two-dimensional SDS-polyacrylamide gel electrophoresis (1D and 2D SDS-PAGE, respectively) as previously described [8]. Protein concentrations were determined using the DC protein assay kit (Bio-Rad). Total protein separated by 1D SDS-PAGE was detected by SYPRO Ruby protein gel stain (Sigma-Aldrich Inc, Ireland) as per manufacturer's instructions. Images were visualized with the BioSpectrum AC Imaging System (Ultra-Violet Products Ltd, UK). For immunoblotting, proteins were transferred to nylon-supported nitrocellulose, incubated with rabbit polyclonal antiserum directed against Sph2 [34], LipL32 [38] and LipL41 [39] followed by goat anti-rabbit secondary antibody (Southern Biotechnology Associates, Birmingham, Ala.). Blots were developed using the SuperSignal West Pico chemiluminescence substrate according to the manufacturer's instructions (Pierce, Rockford, Ill.). 2D gels were loaded with 500 µg total protein and stained with silver as previously described [8].

### RNA isolation, library preparation and RNA-Seq

Total RNA was extracted using TRIzol reagent (Invitrogen) from three biologically-independent samples of (i) *in vitro*-cultivated leptospires or (ii) leptospires cultivated in DMCs (2 rats per sample) for 10 days as described above. Purified RNA was treated with Turbo DNA*free* (Ambion, Inc. Austin, TX) as previously described [108] to remove contaminating genomic DNA. The integrity of DNase-treated RNAs use for RNA-Seq were assessed using the Agilent Bioanalyzer RNA NanoChip (Agilent Technologies, Wilmington, DE) to ensure that each had an RNA integrity (RIN) value ≥8. One-hundred ng of total RNA was used for library generation according to Illumina standard protocols (TruSeq RNA Sample Preparation Guide, Low-Throughput Protocol, Part # 15008136 Rev. A). cDNAs were normalized using a duplex-specific nuclease (DSN) approach according to the DSN Normalization Sample Preparation Guide, Early Access Protocol, Part # 15014673 Rev. C, which decreases the prevalence of highly abundant transcripts, such as rRNAs. 76-bp paired-end sequencing was carried out by Sequensys (Prognosys Biosciences, La Jolla, USA) on an Illumina Genome Analyzer IIx according to the manufacturer's instructions.

### RNA-Seq data analysis

Mapping of sequenced reads to Chromosome 1 and 2 of the reference genome of *Leptospira interrogans* sv. Copenhageni strain Fiocuz L1-130 (NCBI Reference Sequence: NC_005823.1 and NC_005824.1 respectively) [11] was carried out using the software tool segemehl [109] with accuracy set to 100%. To increase coverage, mismatched nucleotides at the lower-quality 3′ end were removed from the reads and the mapping was repeated until a match was found or the read length decreased below 20 nucleotides (see [110]). Reads that mapped to (i) ribosomal or transfer RNAs or (ii) more than one reference genome location (*e.g.*, paralogous genes) were discarded. Uniquely mapped reads (*i.e.*, mapped to a single genomic location) were selected for further analysis, such as data visualisation and determination of differential gene expression. Normalization, differentially-expressed genes, regulatory fold-changes and statistical significance were determined using DESeq [43]. Read coverage used for graphical display was normalized as follows to compensate for different library sizes: the number of reads covering each nucleotide position was divided by the total number of mapped reads in the library and then multiplied with the number of mapped reads from the smallest library. Mapped unique reads were visualised with the Integrated Genome Browser (IGB, version 5.5) (http://bioviz.org/igb/releases.html) [111].

### Bioinformatics

Putative orthologous relations between proteins in other *Leptospira* serovars and/or species were determined using BlastP alignment (≥40% amino acid identify over ≥80% of the length of the smallest protein) as previously described [14]. Protein sequence similarity between differentially-expressed genes identified in *L. interrogans* sv. Copenhageni and other *Leptospira* spp. (*L. interrogans* sv. Lai strain 56601 [80]; *L. borgpetersenii* sv. Hardjo strain L550 [13]; *L. santarosai* sv. Shermani strain LT821 [15]; *L. licerasiae* sv. Varillal strain VAR010 [16]; and *L. biflexa* sv. Patoc strain Patoc1 Ames [14]) was determined using GLSEARCH (version 35.04) from the FASTA package [112]. GLSEARCH identifies the optimal alignment across the entire genome of each strain, translated into all six reading frames, and calculates the percent identity across the whole length of the corresponding sequence. Conserved domain searches were performed on full length protein coding sequences using the NCBI Conserved Domain Database interface [44], [45]. The presence of fur boxes was investigated using the predictive computational tool SLiMSearch [62]. SLiMSearch, which can be used to determine the occurrences of a predefined motif in DNA and protein sequences, makes use of disorder and conservation masking to reduce the number of false positives. The fur box consensus sequence ([GC]AT[AT]AT[GC]AT[AT]AT[GC]AT[AT]AT[GC]) used to search the genome of *Leptospira interrogans* sv. Copenhageni was based on that of [61]. Putative functions of candidate sRNAs were identified by BLAST using the Rfam database, Wellcome Trust Sanger Institute (http://rfam.sanger.ac.uk/).

### Quantitative RT-PCR

DNase-treated RNAs (∼1 µg per sample), isolated from leptospires grown to late-logarithmic phase at 30°C *in vitro* and within DMC, were prepared as described above and converted to cDNA using SuperScript III (Invitrogen) in the presence and absence of reverse transcriptase (RT) according to the manufacturer's instructions. cDNAs were assayed in quadruplicate using iQ Supermix (Bio-Rad) using the primer pairs described in Table S1. For relative quantitation of transcript levels, amplicons corresponding to each gene of interest were cloned into the pCR2.1-TOPO cloning vector (Invitrogen), then purified recombinant plasmid DNAs for each amplicon were diluted (10^7^–10^2^ copies/µl) to generate a standard curve. Reaction conditions for each primer pair were optimized to ensure that each had an amplification efficiency of >90%. Transcript copy numbers for each gene of interest were calculated using the iCycler post-run analysis software based on internal standard curves then normalized against copies of *lipL32* (*LIC11352*) present in the same cDNA. Normalized copy number values were compared within Prism v5.00 (GraphPad Software, San Diego, CA) using an unpaired *t*-test with two-tailed *p* values and a 95% confidence interval.

## Supporting Information

Figure S1 **Comparison of leptospires cultivated** ***in vitro*** **and within DMCs by two dimensional SDS-PAGE revealed numerous polypeptide differences.** Protein lysates prepared from *L. interrogans* sv. Copenhageni strain Fiocruz F1-130 grown at 30°C in EMJH medium (top) or within dialysis membrane chambers (DMCs; bottom). Total protein (500 µg per gel) was solubilized in 7 M urea, 2 M Thiourea and 1% ASB-14 and separated by two-dimensional gel electrophoresis as previously described [8]. Proteins were visualized with Lavapurple.
(TIFF)

Figure S2 **Clustering of biological replicates.** Heatmap representing the expression data for genes whose expression was either positively- or negatively-regulated by ≥Log_2_-fold (adjusted p-value≤0.05) in DMC- versus *in vitro*-cultivated *L. interrogans* sv. Copenhageni strain Fiocruz L1-130.
(TIF)

Figure S3 **IGB viewer of putative sRNAs (** ***LIC1nc10*** **-** ***LIC2nc40*** **) mapping to non-annotated regions of the genome.** Candidate sRNAs were identified as areas of high transcriptional activity in intergenic regions of the genome of *L. interrogans* sv. Copenhageni Fiocruz L1-130. Expression data for leptospires cultivated in DMCs (green) compared to those cultivated *in vitro* (IV, red) are indicated on plus strand of the genome. Annotated genes on the relevant chromosome and nucleotide coordinates are indicated. The vertical “read count” scale is 0–100.
(TIFF)

Figure S4 **Validation of RNA-Seq analysis.** qRT-PCR analysis of the entire panel of genes used to validate RNA-Seq data derived from *L. interrogans* sv. Copenhageni cultivated in EMJH at 30°C *in vitro* (IV) and within DMCs. Values represent the average transcript copy number for each gene normalized per copy of *lipL32*. Bars indicate the standard error of the mean (SEM). Results presented are mean values from at least 3 biologically-independent samples of leptospires for each growth condition. The fold-regulation for each gene determined by RNA-Seq is indicated in parentheses. The folds of regulation between *in vitro*- and DMC-cultivated leptospires determined by qRT-PCR are indicated. P values were calculated using an unpaired *t*-test.
(TIF)

Table S1 **Oligonucleotide primers used in these studies.**
(DOCX)

Table S2 **Gene expression data for Chromosome 1 and 2.** Column A: Gene identification, Column B: Mean number of DESeq values for each gene in all 6 biological replicates, Column C: Mean DESeq values for each gene in 3 biological replicates of leptospires cultured *in vitro* (IV), Column D: Mean DESeq values for each gene in 3 biological replicates of leptospires cultured in dialysis membrane chambers (DMC), Column E: Fold change gene expression by DMC leptospires compared to IV leptospires, Column F: Log_2_ fold change gene expression by DMC leptospires compared to IV leptospires, Column G: p-value of differential gene expression, Column H: adjusted p-value of differential gene expression, Column I: Residual variance of DESeq values for each gene in three biological replicates of IV, Column J: Residual variance of DESeq values for each gene in three biological replicates of DMC, Column L-Q: DESeq values for each gene in each biological replicate, Column S: DNA strand location for each gene, Column T: location of gene on positive or negative strand, Column U: Start position for each gene, Column V: End position for each gene, Column W: gene length, Column X: gene name, Column Y: gene product. Datasheets are arranged to present (1) data for all genes sorted according to the expression values in DMCs; (2) data for all genes sorted according to the expression values *in vitro*; (3) data for genes whose expression was upregulated within DMCs compared to *in vitro*; and (4) data for genes whose expression was downregulated within DMCs compared to *in vitro*.
(XLSX)

Table S3 **Top 100 protein–coding genes expressed by** ***L. interrogans*** **within DMCs.**
(DOCX)

Table S4 **Expression data for individual genes and pathways highlighted in the manuscript.**
(DOCX)

Table S5 **Redox-relevant proteins encoded within the genomes of the pathogenic spirochetes** ***Treponema pallidum*** **,** ***Borrelia burgdorferi*** **and** ***L. interrogans*** **sv. Copenhageni.**
(DOCX)
