# Supplementary figures and images for: A Model System for Studying the Transcriptomic and Physiological Changes Associated with Mammalian Host-Adaptation by Leptospira interrogans Serovar Copenhageni
Source: PLoS Pathog. 2014 Mar 13;10(3):e1004004. doi: 10.1371/journal.ppat.1004004 (PMC3953431; doi:10.1371/journal.ppat.1004004)

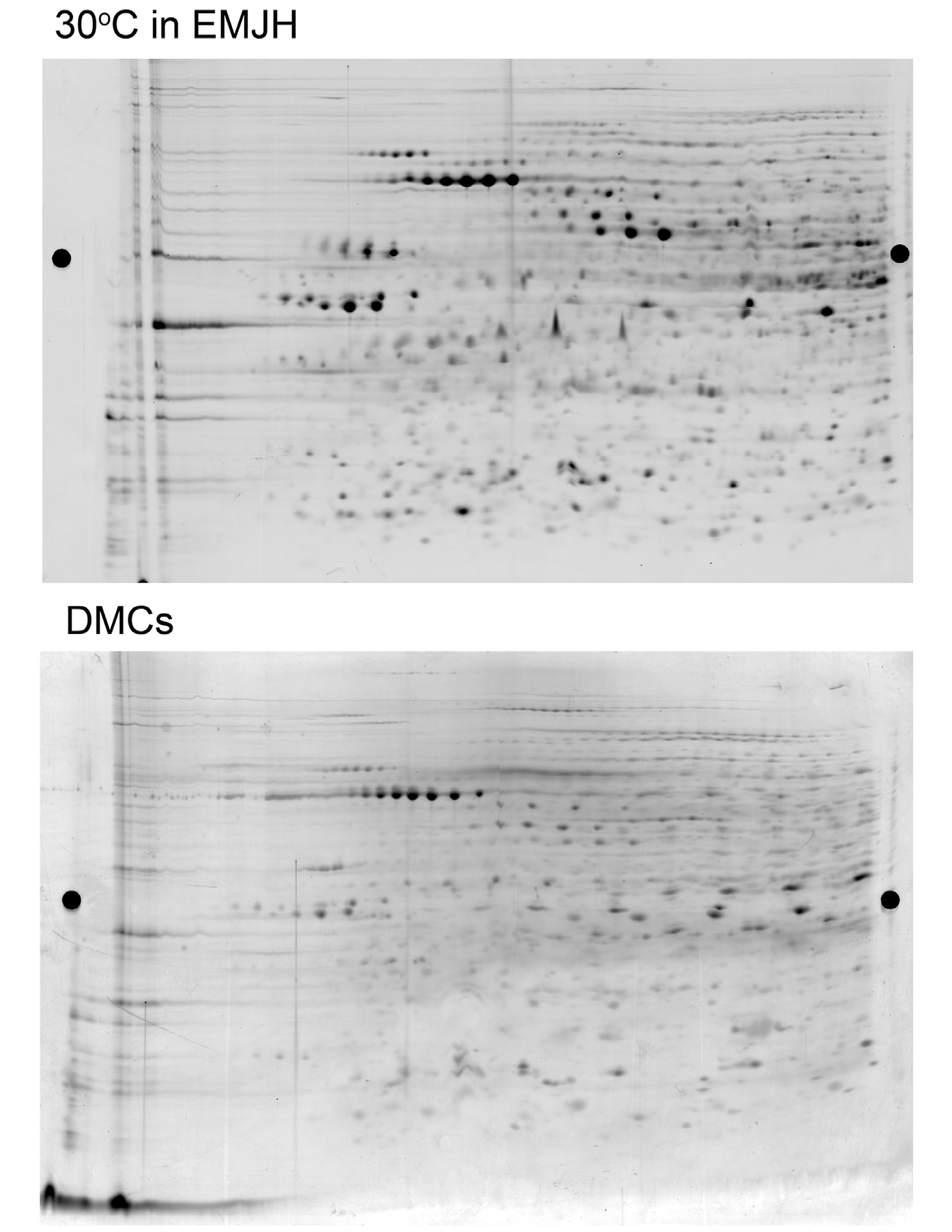

Supplement: Figure S1 — Comparison of leptospires cultivated in vitro and within DMCs by two dimensional SDS-PAGE revealed numerous polypeptide differences. Protein lysates prepared from L. interrogans sv. Copenhageni strain Fiocruz F1-130 grown at 30°C in EMJH medium (top) or within dialysis membrane chambers (DMCs; bottom). Total protein (500 µg per gel) was solubilized in 7 M urea, 2 M Thiourea and 1% ASB-14 and separated by two-dimensional gel electrophoresis as previously described [8]. Proteins were visualized with Lavapurple. (TIFF) [file ppat.1004004.s001.tiff]

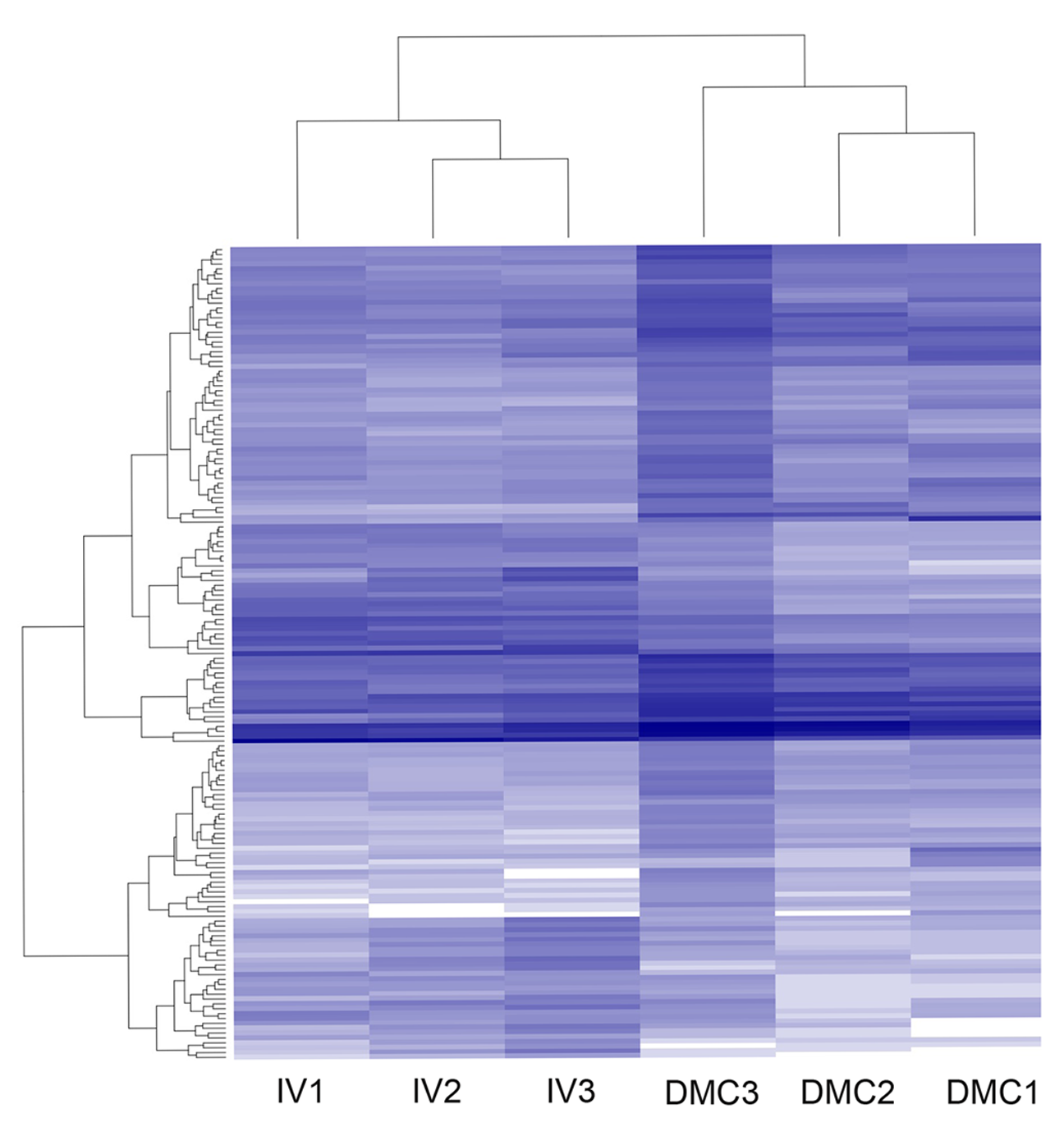

Supplement: Figure S2 — Clustering of biological replicates. Heatmap representing the expression data for genes whose expression was either positively- or negatively-regulated by ≥Log2-fold (adjusted p-value≤0.05) in DMC- versus in vitro-cultivated L. interrogans sv. Copenhageni strain Fiocruz L1-130. (TIF) [file ppat.1004004.s002.tif]

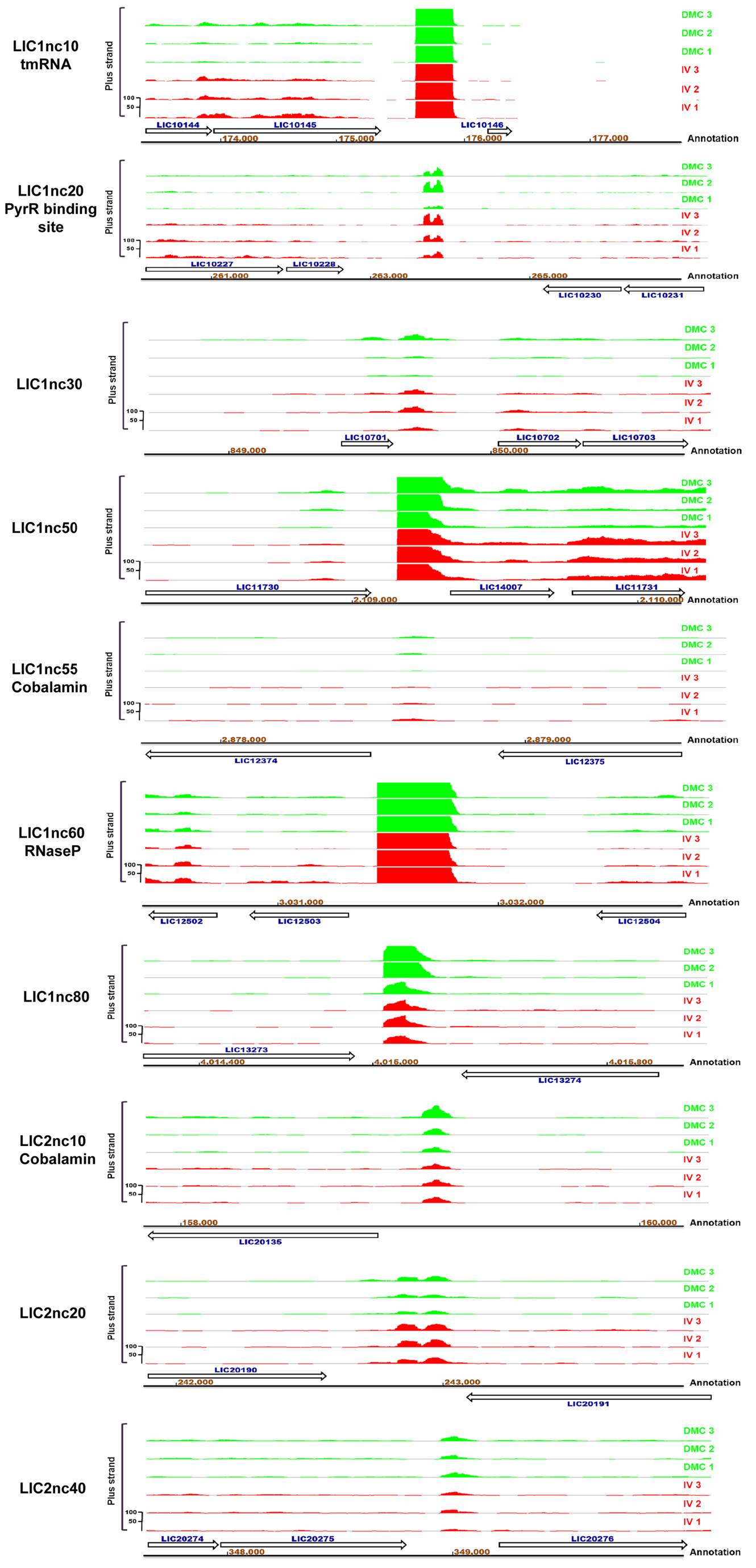

Supplement: Figure S3 — IGB viewer of putative sRNAs ( LIC1nc10 - LIC2nc40 ) mapping to non-annotated regions of the genome. Candidate sRNAs were identified as areas of high transcriptional activity in intergenic regions of the genome of L. interrogans sv. Copenhageni Fiocruz L1-130. Expression data for leptospires cultivated in DMCs (green) compared to those cultivated in vitro (IV, red) are indicated on plus strand of the genome. Annotated genes on the relevant chromosome and nucleotide coordinates are indicated. The vertical “read count” scale is 0–100. (TIFF) [file ppat.1004004.s003.tiff]

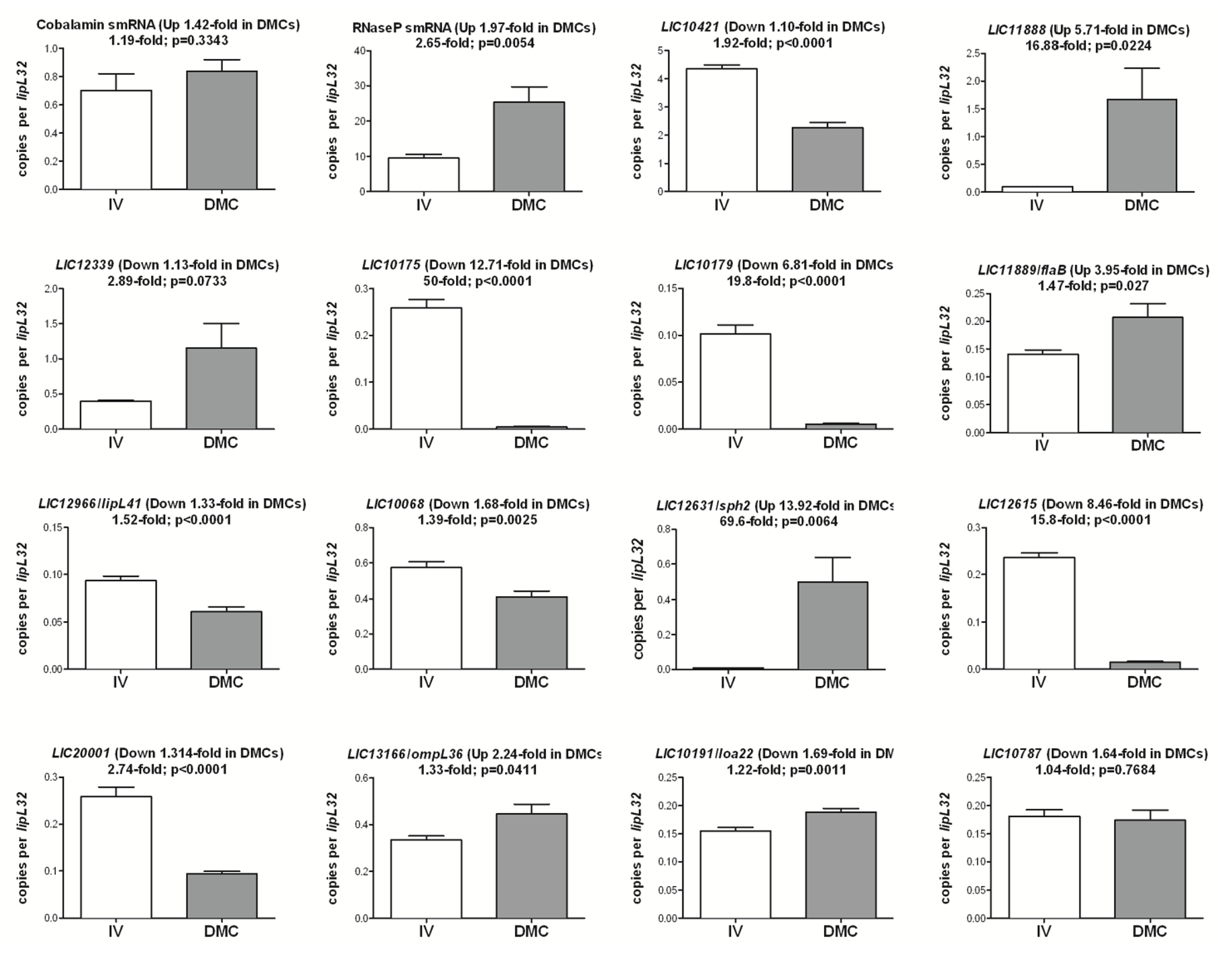

Supplement: Figure S4 — Validation of RNA-Seq analysis. qRT-PCR analysis of the entire panel of genes used to validate RNA-Seq data derived from L. interrogans sv. Copenhageni cultivated in EMJH at 30°C in vitro (IV) and within DMCs. Values represent the average transcript copy number for each gene normalized per copy of lipL32. Bars indicate the standard error of the mean (SEM). Results presented are mean values from at least 3 biologically-independent samples of leptospires for each growth condition. The fold-regulation for each gene determined by RNA-Seq is indicated in parentheses. The folds of regulation between in vitro- and DMC-cultivated leptospires determined by qRT-PCR are indicated. P values were calculated using an unpaired t-test. (TIF) [file ppat.1004004.s004.tif]
