# Supplementary material for: A Model System for Studying the Transcriptomic and Physiological Changes Associated with Mammalian Host-Adaptation by Leptospira interrogans Serovar Copenhageni
Source: PLoS Pathog. 2014 Mar 13;10(3):e1004004. doi: 10.1371/journal.ppat.1004004 (PMC3953431; doi:10.1371/journal.ppat.1004004)
Supplement: Table S1 — Oligonucleotide primers used in these studies. (DOCX) [file ppat.1004004.s005.docx]

**Supplemental Table 1. Oligonucleotide primers used in these studies**

| **Gene ID** | **Description** | **Forward Primer (5’-3’)** | **Position^1^** | **Reverse Primer (5’-3’)** | **Position^1^** |
| --- | --- | --- | --- | --- | --- |
| LIC10068 | Hypothetical protein | AAGGAGTCAAGGATACAA | 87420 | TATAGAAATCCCCGTCTT | 87554 |
| LIC10175 | Hypothetical protein | AGGTAGTAGAGTTGTAGTA | 203587 | GAGTAGAAGCATTCGTAA | 203762 |
| LIC10179 | Hypothetical protein | AATGCTCCGATTCAATATG | 208444 | AAGAAGACTCCACTACAC | 208555 |
| LIC10191 | Loa22 | GATAGTTACGCTCTTGAA | 221020 | GATACGATTTGCTGGAAT | 220868 |
| LIC10421 | Hypothetical protein | TTTATCTGTAGGTTCTCTATTTG | 481754 | ATTTCCAATCGCAGTCTT | 481865 |
| LIC10787 | FlaA flagellar filament sheath protein | CGGAAATAATCAGGCTGGTGG | 954390 | TGTTTTCGYCGAACACGTCTC | 954574 |
| LIC11352 | LipL32 | CTGAGCGAGGACACAATC | 1667015 | ATTACGGCAGGAATCCAA | 1666849 |
| LIC11888 | Flagellar hook protein | TTCGTCAATATAGAGGCATA | 2284844 | AATAGAGTCCCACCAAAG | 2284658 |
| LIC11889 | FlaB flagellin | CTGAAGACGGAATGAGTT | 2287024 | CGGATTCTTTGAATGATGTT | 2286949 |
| LIC12339 | Hypothetical protein | AACACTTACAACCATACG | 2827297 | ATATCTTCCTGAACCTCTT | 2827186 |
| LIC12615 | Hypothetical protein | CGGATGTTGTACCTTATAC | 3163364 | GCGATGATACCTTGAATT | 3163271 |
| LIC12631 | Sphingomyelinase-like protein Sph2 | AGTTGTAATCCTAAGTAAATGG | 3184191 | TTCTGATCCTGAGACTGA | 3184361 |
| LIC12966 | LipL41 | CGTATGATGTTAATTCCTCTC | 3604118 | AAACCTTGTCCGAAAGAA | 3604266 |
| LIC13166 | OmpL36 | AAGAGCAGAAGCGTCGTA | 3876182 | TTGGAGAGTTGGTGGAGTT | 3876290 |
| LIC20001 | Hypothetical protein | CTTATCCTGGAATTGTAGAAT | 1591 | AATCGTGAGTATGAGGTT | 1707 |
| **Putative Small RNAs (quantitative RT-PCR)** | |  | |  | |
| LIC1nc60 | RNaseP | AAAAGGGTGAGGAAAGTC | 3031494 | TCTTTTCTGTTGCACTT | 3031601 |
| LIC2nc10 | Cobalamin | TCATAGGTCGTATTCTGA | 159093 | AAGACATACTAAGCCACT | 159169 |
| **Putative Small RNAs (validation)** | |  |  | | |
| LIC1nc10 | tmRNA | AGCAGCTTAATCTCTGCTACGG | 175718 | CCCTCAGACAGGACTCTCTTTC | 175863 |
| LIC1nc20 | PyrR | GGAGTTAACTTGACCCGCAAC | 263777 | CACTTCGAGAGCAAGCAGACAG | 263940 |
| LIC1nc30 |  | TTCGCAGTAGTTCCCACATTTG | 849688 | TCTGAGCCTACAGGTCGATTCC | 849794 |
| LIC1nc50 |  | CCGGGCTCTTTGCAGGAATG | 2109169 | GGACGGAATCCGAACCATCATC | 2109308 |
| LIC2nc10 | Cobalamin | CGAGGTCGAAACGCACTTTCAG | 159065 | AAAGACATACTAAGCCACTGGG | 159170 |
| LIC2nc20 |  | CATAGTTGCGATGTTTGCTGCG | 242924 | CATTGACCGTAATGTGTGGAGG | 243048 |
| LIC2nc30 |  | AGAAACTCAGAAAGTTCAGCGG | 246213 | CTGTGAGGTGGCTGAGCAATAG | 246309 |

^1^ Chromosomal coordinates for forward and reverse primers
