# Supplementary material for: A Model System for Studying the Transcriptomic and Physiological Changes Associated with Mammalian Host-Adaptation by Leptospira interrogans Serovar Copenhageni
Source: PLoS Pathog. 2014 Mar 13;10(3):e1004004. doi: 10.1371/journal.ppat.1004004 (PMC3953431; doi:10.1371/journal.ppat.1004004)
Supplement: Table S3 — Top 100 protein–coding genes expressed by L. interrogans within DMCs. (DOCX) [file ppat.1004004.s007.docx]

**Table S3. Top 100 protein–coding genes expressed by *L. interrogans* within DMCs.**

| Gene ID | Gene name | Orthologous^1^ | Annotation | DMC^2^ | IV^2^ | Fold-Change | P-adj^3^ |
| --- | --- | --- | --- | --- | --- | --- | --- |
| LIC10524 | *dnaK* | All | Molecular chaperone DnaK | 3,472.34 | 1,741.08 | 1.99 | 0.711 |
| LIC10464 | *ligB* | 1,2,3,4 | Hypothetical protein | 3,447.50 | 1,125.45 | 3.06 | 0.254 |
| LIC12210 |  | 1,2,3,4 | Hsp15-like protein | 3,215.70 | 1,191.04 | 2.70 | 0.442 |
| LIC12297 |  | 1,2,3,4 | **DNA repair protein** | **3,157.27** | **693.81** | **4.55** | **0.038** |
| LIC12298 |  | 2,3,4,5 | Hypothetical protein | 2,801.05 | 730.57 | 3.83 | 0.076 |
| LIC10465 | ***ligA*** | **1** | ***Leptospira* Ig-like protein LigA** | **2,633.11** | **576.22** | **4.57** | **0.020** |
| LIC11352 | *lipL32* | 1,2,3,4 | Hypothetical protein | 2,625.31 | 2,744.30 | -1.04 | 0.985 |
| LIC11335 | *groEL* | All | Chaperonin GroEL | 2,577.19 | 958.88 | 2.69 | 0.328 |
| LIC12211 | *hsp15* | 1,2,3,4 | Hsp15 | 1,938.45 | 653.11 | 2.97 | 0.215 |
| LIC11889 | ***flaB*** | **All** | **Flagellin protein** | **1,761.90** | **446.34** | **3.95** | **0.015** |
| LIC10339 | *tlyB* | All | Hemolysin B | 1,706.22 | 1,348.30 | 1.27 | 0.906 |
| LIC12339 |  | 1,2,3 | Hypothetical protein | 1,590.15 | 1,403.84 | 1.13 | 0.996 |
| LIC11890 |  | All | Flagellin protein | 1,562.75 | 699.43 | 2.23 | 0.380 |
| LIC12032 | *katE* | 1,2,3 | Catalase | 1,363.79 | 761.75 | 1.79 | 0.681 |
| LIC13166 |  | All | Hypothetical protein | 1,309.11 | 585.01 | 2.24 | 0.342 |
| LIC10123 |  | 1,2,3,4 | Hypothetical protein | 1,299.79 | 799.52 | 1.63 | 0.763 |
| LIC12324 |  | 1,2,4 | Serine/threonine kinase protein | 1,221.98 | 647.82 | 1.89 | 0.437 |
| LIC10753 | *rpoB* | All | DNA-directed RNA polymerase subunit β | 1,216.29 | 1,654.50 | -1.35 | 0.846 |
| LIC10754 | *rpoC* | All | DNA-directed RNA polymerase subunit β’ | 1,202.17 | 1,875.83 | -1.56 | 0.731 |
| LIC12875 | *tuf* | All | Elongation factor Tu | 1,183.25 | 1,414.55 | -1.19 | 0.902 |
| LIC11587 | *nrdA* | All | Ribonucleotide-diphosphate reductase subunit α | 987.63 | 1,841.81 | -1.85 | 0.533 |
| LIC10874 |  | All | Molybdopterin oxidoreductase | 924.11 | 1,299.99 | 0.71 | 0.834 |
| LIC12631 | ***sph2*** | **1,2** | **Hemolysin** | **908.90** | **65.30** | **13.92** | **5.13×10^-26^** |
| LIC12979 |  | 1,2,3 | Hypothetical protein | 900.19 | 515.27 | 1.75 | 0.562 |
| LIC12227 |  | 1,4,5 | Hypothetical protein | 876.18 | 1,027.32 | -1.18 | 0.866 |
| LIC20083 | *ahcY* | All | S-adenosyl-L-homocysteine hydrolase | 874.27 | 627.97 | 1.39 | 0.884 |
| LIC11219 | ***ahpC*** | **All** | **Peroxiredoxin** | **859.87** | **144.23** | **5.96** | **7.29×10^-7^** |
| LIC20141 | *htpX* | All | Heat shock protein HtpX | 812.39 | 1,029.75 | -1.70 | 0.731 |
| LIC20249 | *acnA* | All | Aconitate hydratase | 806.90 | 866.95 | -1.07 | 0.930 |
| LIC10421 |  | 1,2,3,4 | Hypothetical protein | 804.59 | 889.32 | -1.11 | 0.872 |
| LIC11745 | *recA* | All | Recombinase A | 799.82 | 675.05 | 1.18 | 0.954 |
| LIC10244 |  | 1,2,3 | Hypothetical protein | 785.31 | 547.89 | 1.43 | 0.747 |
| LIC10970 |  | All | Acyl-CoA dehydrogenase | 775.31 | 395.21 | 1.96 | 0.502 |
| LIC10789 | *greA* | All | Transcript cleavage factor/unknown domain fusion protein | 769.71 | 890.38 | -1.16 | 0.959 |
| LIC12653 |  | **Unique** | **Hypothetical protein** | **752.08** | **207.47** | **3.63** | **0.0058** |
| LIC10068 |  | 1,2,3,4 | Hypothetical protein | 746.17 | 1,257.81 | -1.69 | 0.517 |
| LIC11643 | *lipL45* | All | Hypothetical protein | 733.56 | 384.35 | 1.91 | 0.408 |
| LIC12228 |  | 1,4 | Hypothetical protein | 715.88 | 659.58 | 1.09 | 1.000 |
| LIC11709 |  | 1,2,3,4 | Histidine kinase response regulator hybrid protein | 711.46 | 446.29 | 1.59 | 0.558 |
| LIC12317 | *sch1* | 1,2 | β-ketoacyl synthase | 706.06 | 760.64 | -1.08 | 0.959 |
| LIC20078 |  | 1,2,3,4 | Hypothetical protein | 704.50 | 287.49 | 2.45 | 0.166 |
| LIC11149 |  | All | Membrane metalloendopeptidase | 678.76 | 394.39 | 1.72 | 0.417 |
| LIC10525 | *grpE* | All | Heat shock protein GrpE | 678.20 | 288.54 | 2.35 | 0.227 |
| LIC10272 | *fusA* | All | Elongation factor G | 669.08 | 591.33 | 1.13 | 0.983 |
| LIC10011 | *lipL21* | All | Hypothetical protein | 667.80 | 669.36 | 1.00 | 1.000 |
| LIC12705 | *infB* | 1,2,3,4 | Translation initiation factor IF-2 | 639.15 | 827.75 | -1.30 | 0.888 |
| LIC12966 | *lipL41* | 1,2,3,4 | Hypothetical protein | 617.11 | 822.14 | -1.33 | 0.740 |
| LIC10523 | *dnaJ* | All | Chaperone protein DnaJ | 615.74 | 390.51 | 1.58 | 0.568 |
| LIC11463 |  | 1,2,3 | Hypothetical protein | 610.53 | 336.38 | 1.82 | 0.458 |
| LIC10191 |  | All | Hypothetical protein | 600.13 | 352.59 | 1.70 | 0.481 |
| LIC12407 | *glnA* | All | Glutamine synthetase protein | 590.65 | 457.75 | 1.29 | 0.785 |
| LIC11531 |  | All | Flagellin protein | 587.65 | 359.88 | 1.63 | 0.553 |
| LIC11851 | *impL63* | All | Hypothetical protein | 570.14 | 270.65 | 2.11 | 0.117 |
| LIC11617 |  | **1,2,4,5** | **ArsR family transcriptional regulator** | **569.66** | **183.09** | **3.11** | **0.0073** |
| LIC13050 |  | 1,2,3,4 | Hypothetical protein | 567.24 | 457.67 | 1.24 | 0.886 |
| LIC12002 | *sdhA* | All | Succinate dehydrogenase flavoprotein subunit | 566.26 | 629.78 | -1.11 | 0.877 |
| LIC12412 |  | All | Chromosome segregation protein | 563.52 | 600.30 | -1.06 | 0.959 |
| LIC10973 | *ompL1* | All | Outer membrane protein | 558.75 | 540.62 | 1.03 | 1.000 |
| LIC11888 |  | **1,2,3** | **Hypothetical protein** | **550.35** | **96.44** | **5.71** | **1.13×10^-8^** |
| LIC12701 | *pnpA* | All | Polynucleotide phosphorylase/polyadenylase | 511.60 | 795.64 | -1.56 | 0.785 |
| LIC10723 | *fliD* | All | Flagellar hook-associated protein FliD | 510.30 | 294.88 | 1.73 | 0.461 |
| LIC12474 | *sucA* | All | 2-oxoglutarate dehydrogenase E1 component | 505.45 | 466.18 | 1.08 | 1.000 |
| LIC12447 | *rpsA* | All | 30S ribosomal protein S1 | 505.24 | 556.41 | -1.10 | 0.940 |
| LIC20052 | *scd* | 1,2,3,4 | Fatty acid desaturase | 495.92 | 540.13 | -1.09 | 0.959 |
| LIC10788 | *flaA-1* | All | Flagellar filament sheath protein | 494.55 | 339.22 | 1.46 | 0.704 |
| LIC11630 | *fadD* | All | Long-chain-fatty-acid CoA ligase | 492.84 | 722.30 | -1.47 | 0.699 |
| LIC12397 |  | All | Hypothetical protein | 488.53 | 270.54 | 1.81 | 0.375 |
| LIC11336 | *groES* | All | Co-chaperonin GroES | 488.12 | 229.07 | 2.13 | 0.224 |
| LIC10816 |  | 1,2,3 | Hypothetical protein | 486.93 | 1,004.74 | -2.08 | 0.289 |
| LIC10358 |  | All | Hypothetical protein | 485.86 | 228.72 | 2.12 | 0.107 |
| LIC10209 | *cyoB* | All | Cytochrome c oxidase polypeptide I | 484.46 | 571.95 | -1.18 | 0.889 |
| LIC12760 |  | **1,2,4** | **Collagenase precursor** | **475.22** | **9.69** | **49.03** | **2.63×10^-51^** |
| LIC12322 |  | **1,2,4** | **Glutaconate CoA transferase-like protein** | **464.64** | **200.53** | **2.32** | **0.048** |
| LIC10371 |  | **1,2,3** | **Lipoprotein** | **459.23** | **99.70** | **4.61** | **0.000** |
| LIC11729 | *fadH* | All | 2,4-dienoyl-coa reductase | 455.95 | 494.16 | -1.09 | 0.969 |
| LIC12812 | *mucD* | 1,2,4,5 | Serine protease MucD precursor | 452.04 | 496.51 | -1.10 | 0.848 |
| LIC13046 |  | 1,2,3,5 | UDP glucosamine N-acyltransferase | 440.93 | 367.66 | 1.20 | 0.894 |
| LIC11633 | *lpxC* | All | UDP-3-O-[3-hydroxymyristoyl] N-acetylglucosamine deacetylase | 440.06 | 388.45 | 1.13 | 0.740 |
| LIC10216 | *pckA* | All | Phosphoenolpyruvate carboxykinase | 438.80 | 628.29 | -1.43 | 0.712 |
| LIC11241 | *atpA* | All | F_0_F_1_ ATP synthase subunit α | 432.98 | 332.38 | 1.30 | 0.877 |
| LIC12587 |  | 1,2,3,4 | Lipoprotein | 428.71 | 250.62 | 1.71 | 0.472 |
| LIC12017 | ***clpB*** | **All** | **ATP-dependent protease** | **427.28** | **2,010.52** | **-4.76** | **0.011** |
| LIC11407 |  | 1,2,3,4 | Methyl-accepting chemotaxis transmembrane protein | 425.94 | 248.67 | 1.71 | 0.385 |
| LIC12706 | *nusA* | All | Transcription elongation factor NusA | 425.06 | 509.05 | -1.20 | 0.943 |
| LIC10538 | *ybiT* | All | ABC transporter ATP-binding protein | 423.93 | 240.12 | 1.77 | 0.387 |
| LIC20250 |  | 1,2,3 | Peptidoglycan-associated cytoplasmic membrane protein | 416.72 | 420.43 | -1.01 | 0.966 |
| LIC11977 |  | All | Cyclic nucleotide binding protein | 415.89 | 373.62 | 1.11 | 0.903 |
| LIC10988 |  | 1,2,3 | Hypothetical protein | 414.17 | 331.76 | 1.25 | 0.848 |
| LIC11532 |  | All | Flagellin protein | 393.28 | 275.21 | 1.43 | 0.727 |
| LIC11848 |  | All | Hypothetical protein | 392.08 | 263.20 | 1.49 | 0.662 |
| LIC20001 |  | All | Hypothetical protein | 387.90 | 550.50 | -1.43 | 0.735 |
| LIC11944 | *secA* | All | Preprotein translocase subunit SecA | 387.17 | 354.63 | 1.09 | 0.956 |
| LIC20085 | *metH* | All | B_12_-dependent methionine synthase | 382.31 | 340.99 | 1.12 | 0.940 |
| LIC10526 | *hrcA* | All | Heat-inducible transcriptional repressor | 371.17 | 224.78 | 1.65 | 0.490 |
| LIC13348 | *gyrB* | All | DNA gyrase subunit B | 370.03 | 331.63 | 1.12 | 0.961 |
| LIC12273 |  | All | GGDEF family protein | 367.76 | 390.71 | -1.06 | 0.969 |
| LIC10222 | *dnaE* | All | DNA polymerase III subunit alpha | 362.50 | 467.79 | -1.30 | 0.837 |
| LIC10750 | *rplA* | All | 50S ribosomal protein L1 | 362.08 | 359.34 | 1.01 | 1.000 |
| LIC11442 |  | 1,2,3,5 | Hypothetical protein | 360.04 | 418.02 | -1.16 | 0.848 |
| LIC10850 | *rpsB* | All | 30S ribosomal protein S2 | 348.74 | 369.36 | -1.06 | 0.981 |

^1^Putative ortholog present in other *Leptospira* spp. based on a BlastP alignment threshold and a minimum of 40% amino acid sequence identity over ≥80% of the length of the smallest coding region. 1, *L. interrogans* sv. Lai strain 56601 and/or IPAV; 2, *L. borgpetersenii* sv. Hardjo strain L550 and/or JB197; 3, *L. licerasiae* sv. Varillal strain VAR010 and/or MMD0835; 4, *L. santarosai* sv. Shermani strain LT821; and 5, *L. biflexa* sv. Patoc strain Patoc1 Ames and/or Paris; All, represented in at least one strain for all 5 serovars; and Unique, found only in *L. interrogans* sv. Copenhageni strain Fiocruz L1-130.

^2^ Mean values per gene from three biological replicates in either DMC or *in vitro* (IV) conditions (see Table S2).

^3^ Adjusted P-value (see Table S2).

Genes that are differentially-expressed (>2-fold; p≤0.05) by leptospires in DMCs compared to *in vitro* are highlighted in **Bold**.
