# Supplementary material for: A Model System for Studying the Transcriptomic and Physiological Changes Associated with Mammalian Host-Adaptation by Leptospira interrogans Serovar Copenhageni
Source: PLoS Pathog. 2014 Mar 13;10(3):e1004004. doi: 10.1371/journal.ppat.1004004 (PMC3953431; doi:10.1371/journal.ppat.1004004)
Supplement: Table S4 — Expression data for individual genes and pathways highlighted in the manuscript. (DOCX) [file ppat.1004004.s008.docx]

**Table S4. Expression data for individual genes and pathways highlighted in the manuscript.**

**A. Uptake and β-oxidation of long chain and unsaturated fatty acids (KEGG pathway lic00010)**

| LIC ID |  | Gene | Product | DMC^1^ | IV^1^ | Fold-change | P-adj^2^ |
| --- | --- | --- | --- | --- | --- | --- | --- |
| 12524 | FadL | *salD/fadL* | fatty acid transport protein | 7.71 | 4.77 | 1.62 | 0.83 |
| 11630 | FadD | *fadD* | long-chain-fatty-acid CoA ligase/acyl-CoA synthase | 492.84 | 722.30 | -1.47 | 0.70 |
| 10094 | (4 paralogs) |  |  | 326.90 | 246.96 | 1.32 | 0.729 |
| 11747 |  | *ydiD* |  | 60.00 | 74.26 | -1.23 | 0.834 |
| 20074 |  |  |  | 88.15 | 127.23 | -1.45 | 0.433 |
| 12551 | FadE-1 | *acd3* | acyl-CoA dehydrogenase | 41.82 | 48.86 | -1.17 | 0.87 |
| 10583 | (5 paralogs) |  |  | 47.11 | 84.63 | -1.79 | 0.347 |
| 11350 |  |  |  | 52.39 | 49.85 | 1.05 | 0.996 |
| 11976 |  |  |  | 193.80 | 218.15 | -1.12 | 0.943 |
| 13281 |  |  |  | 48.29 | 53.84 | -1.11 | 0.956 |
| 13136 | FadE2 | *acd* | acyl-CoA dehydrogenase | 47.89 | 46.70 | 1.03 | 0.98 |
| 10066 | FadB | *ech1* | enoyl-CoA hydratase/isomerase family protein | 28.81 | 21.86 | 1.32 | 0.63 |
| 12725 | (8 paralogs) | *ech2* | 3-hydroxybutyryl-CoA dehydratase | 50.67 | 43.08 | 1.18 | 0.73 |
| 12629 |  | *ech3* | enoyl-CoA hydratase | 210.09 | 125.21 | 1.68 | 0.39 |
| 12495 |  | *ech4/crt* | 3-hydroxybutyryl-CoA dehydratase | 79.51 | 88.42 | -1.11 | 0.87 |
| 11672 |  | *ech5* | enoyl-CoA hydratase | 20.94 | 23.11 | -1.10 | 0.94 |
| 10799 |  | *ech6* | enoyl-CoA hydratase | 16.43 | 7.58 | 2.17 | 0.21 |
| **10565** |  | ***hbd1*** | **enoyl-CoA hydratase** | **10.51** | **34.46** | **-3.28** | **0.02** |
| 13300 |  | *had/fadB* | 3-hydroxyacyl-CoA dehydrogenase | 89.68 | 152.87 | -1.70 | 0.23 |
| 11311 | FadA | *thl1/erg10* | acetyl-CoA C-acyltransferase | 96.62 | 70.09 | 1.38 | 0.56 |
| 12795 | (4 paralogs) | *thl2* |  | 348.22 | 228.00 | 1.53 | 0.83 |
| 10974 |  | *thl4* |  | 104.60 | 99.24 | 1.05 | 0.95 |
| 13301 |  | *thl5* |  | 149.86 | 274.33 | -1.83 | 0.21 |
| 11729 | FadH | *fadH* | 2,4-dienoyl-CoA reductase | 455.95 | 494.16 | -1.09 | 0.969 |

**B. Glycolosis and glycerol utilization (KEGG pathway lic100010)**

| LIC ID | Gene | Product | DMC^1^ | IV^1^ | Fold-change | P-adj^2^ |  |
| --- | --- | --- | --- | --- | --- | --- | --- |
| 12908 | | *sglT* | sodium/glucose cotransport protein | 11.82 | 9.46 | 1.25 | 0.877 |
| 12312 | | *glcK* | glucokinase | 14.45 | 10.23 | 1.41 | 0.729 |
| 13105 | | *pgiB* | glucose-6-phosphate isomerase | 43.04 | 28.31 | 1.52 | 0.59 |
| 11707 | | *fbp* | fructose-1,6-bisphosphatase | 25.18 | 26.70 | -1.06 | 0.848 |
| 20088 | | *pfp* | diphosphate--fructose-6-phosphate 1-phosphotransferase | 47.98 | 43.37 | 1.11 | 1.000 |
| 12233 | | *fbaB* | fructose-bisphosphate aldolase | 90.26 | 120.10 | -1.33 | 0.56 |
| 12094 | | *tpiA* | triosephosphate isomerase | 34.08 | 40.02 | -1.17 | 0.86 |
| 12090 | | *gapA* | glyceraldehyde-3-phosphate dehydrogenase | 201.96 | 291.14 | -1.44 | 0.57 |
| 12091 | | *pgk* | phosphoglycerate kinase | 190.28 | 281.95 | -1.48 | 0.61 |
| 13358 | | *gpmB* | phosphoglycerate mutase | 2.66 | 0.52 | 5.16 | 0.26 |
| 10383 | | *yibO* | phosphoglycerate mutase | 40.91 | 70.10 | -1.71 | 0.223 |
| 20119 | | *pgmA* | phosphoglycerate mutase | 4.09 | 5.48 | -1.34 | 1.000 |
| 11766 | | *manB/pgm* | phosphomannomutase | 25.45 | 25.40 | 1.00 | 0.96 |
| 11954 | | *eno* | phosphopyruvate hydratase | 116.99 | 113.04 | 1.03 | 0.98 |
| 11132 | | *pykA* | pyruvate kinase | 55.13 | 55.65 | -1.01 | 1.00 |
| 20264 | | *pykF* | pyruvate kinase | 33.64 | 44.93 | -1.34 | 0.62 |
| 10216 | | *pckA* | phosphoenolpyruvate carboxykinase | 438.80 | 628.29 | -1.43 | 0.71 |

| 10722 | *glpF* | glycerol uptake facilitator protein | 14.12 | 14.35 | -1.02 | 0.95 |
| --- | --- | --- | --- | --- | --- | --- |
| 11799 | *glpK-1* | glycerol kinase | 34.51 | 57.36 | -1.66 | 0.47 |
| 10629 | *glpK-2* | glycerol kinase | 8.08 | 14.01 | -1.73 | 0.73 |
| 11675 | *gpsA-1* | glycerol-3-phosphate dehydrogenase | 32.60 | 77.13 | -2.37 | 0.17 |
| 12563 | *gspA-2* | glycerol-3-phosphate dehydrogenase | 73.70 | 78.29 | -1.06 | 0.90 |
| 11699 | *glpD* | glycerol-3-phosphate dehydrogenase | 104.24 | 117.42 | -1.13 | 0.83 |
| 13145 | *gpsA* | NAD(P)H-dependent glycerol-3-phosphate dehydrogenase | 48.53 | 42.31 | 1.15 | 0.96 |

**C. Citric acid cycle (KEGG pathway lic00020)**

| LIC ID | Gene | | Product | DMC^1^ | IV^1^ | Fold-change | P-adj^2^ |
| --- | --- | --- | --- | --- | --- | --- | --- |
| 12829 | | *gltA* | citrate synthase | 157.11 | 130.66 | 1.20 | 0.860 |
| 12925 | | gltA | citrate synthase | 55.10 | 60.28 | -1.09 | 1.000 |
| 20249 | | *acn* | aconitate hydratase | 806.90 | 866.95 | -1.07 | 0.93 |
| 13244 | | *icd-1* | isocitrate dehydrogenase | 199.47 | 189.47 | 1.05 | 0.99 |
| 13328 | | *icd-2* | isocitrate dehydrogenase | 57.80 | 69.80 | -1.21 | 0.79 |
| 12474 | | *sucA* | 2-oxoglutarate dehydrogenase E1 component | 505.45 | 466.18 | 1.08 | 1.00 |
| 12573 | | *sucC* | succinyl-CoA synthetase subunit beta | 118.91 | 143.36 | -1.21 | 0.84 |
| 12574 | | *sucD* | succinyl-CoA synthetase subunit alpha | 82.83 | 96.05 | -1.16 | 1.00 |
| 12001 | | *sdhC* | succinate dehydrogenase cytochrome subunit B | 193.54 | 176.77 | 1.09 | 1.000 |
| 12003 | | *sdhB* | succinate dehydrogenase/fumarate reductase iron-sulfur subunit | 323.88 | 293.13 | 1.10 | 0.98 |
| 12002 | | *sdhA* | succinate dehydrogenase flavoprotein subunit | 566.26 | 629.78 | -1.11 | 0.88 |
| 10162 | | *fum* | fumarate hydratase | 66.46 | 77.78 | -1.17 | 0.84 |
| 11781 | | *mdh* | malate dehydrogenase | 292.41 | 339.20 | -1.16 | 0.78 |
| 12476 | | *sucB* | dihydrolipoamide succinyltransferase | 208.57 | 219.43 | -1.05 | 0.969 |
| 11159 | |  | dihydrolipoamide dehydrogenase | 40.95 | 47.34 | -1.16 | 0.817 |
| 11803 | |  | dihydrolipoamide dehydrogenase | 10.26 | 19.10 | -1.85 | 0.224 |
| 12475 | | *lpdA* | dihydrolipoamide dehydrogenase | 220.92 | 229.61 | -1.04 | 1.000 |
| 11897 | | *aceEA/acoA* | pyruvate dehydrogenase alpha2 subunit protein | 52.53 | 60.43 | -1.15 | 0.94 |
| 11898 | | *aceEB/acoB* | pyruvate dehydrogenase subunit beta | 48.25 | 45.64 | 1.06 | 0.97 |
| 11605 | | *aceEF* | dihydrolipoamide acetyltransferase | 5.36 | 9.81 | -1.83 | 0.56 |

**D. Oxidation phosphorylation (KEGG pathway lic10090)**

| LIC ID | Gene | Product | DMC^1^ | IV^1^ | Fold-change | P-adj^2^ |
| --- | --- | --- | --- | --- | --- | --- |
| 12741 | *nuoA* | NADH dehydrogenase subunit I A | 33.64 | 51.57 | -1.53 | 0.73 |
| 12742 | *nuoB* | NADH dehydrogenase subunit B | 66.33 | 67.46 | -1.02 | 0.99 |
| 12743 | *nuoC* | NADH dehydrogenase subunit I C | 39.80 | 35.71 | 1.11 | 0.84 |
| 12744 | *nuoD2* | NADH dehydrogenase subunit I D | 75.55 | 92.22 | -1.22 | 0.97 |
| 12745 | *nuoE* | NADH dehydrogenase subunit I E | 19.20 | 38.18 | -1.99 | 0.38 |
| 12746 | *nuoF* | NADH dehydrogenase subunit I F | 103.93 | 126.04 | -1.21 | 0.83 |
| 10141 | *nuoG* | NADH dehydrogenase subunit I G | 124.53 | 145.32 | -1.17 | 0.87 |
| 12747 | *nuoH* | NADH dehydrogenase subunit I H | 42.13 | 49.37 | -1.17 | 1.00 |
| 10142 | *nuoI* | NADH dehydrogenase II subunit I | 56.10 | 54.77 | 1.02 | 1.00 |
| 12748 | *nuoJ* | NADH dehydrogenase I subunit J | 24.32 | 30.24 | -1.24 | 0.97 |
| 12749 | *nuoK* | NADH dehydrogenase I subunit K | 7.17 | 9.75 | -1.36 | 1.00 |
| 12750 | *nuoL* | NADH dehydrogenase I subunit L | 59.70 | 71.89 | -1.20 | 1.00 |
| 12751 | *nuoM* | NADH dehydrogenase I subunit M | 48.18 | 43.20 | 1.12 | 0.74 |
| 12752 | *nuoN* | NADH dehydrogenase I subunit N | 43.28 | 37.95 | 1.14 | 0.73 |
| 20028 | *ndh* | NADH dehydrogenase II | 115.26 | 171.02 | -1.48 | 0.44 |
| 12001 | *sdhC* | succinate dehydrogenase cytochrome subunit B | 193.54 | 176.77 | 1.09 | 1.000 |
| 12003 | *sdhB* | succinate dehydrogenase/fumarate reductase iron-sulfur subunit | 323.88 | 293.13 | 1.10 | 0.98 |
| 12002 | *sdhA* | succinate dehydrogenase flavoprotein subunit | 566.26 | 629.78 | -1.11 | 0.88 |
| 11237 | *atpB* | F_0_F_1_ ATP synthase subunit A | 85.83 | 52.26 | 1.64 | 0.351 |
| 11238 | *atpE* | ATP synthase C chain | 211.68 | 174.35 | 1.21 | 0.947 |
| 11239 | *atpF* | F_0_F_1_ ATP synthase subunit B | 237.63 | 211.07 | 1.13 | 1.000 |
| 11240 | *atpH* | F_0_F_1_ ATP synthase subunit delta | 98.75 | 98.01 | 1.01 | 0.986 |
| 11241 | *atpA* | F_0_F_1_ ATP synthase subunit alpha | 432.98 | 332.38 | 1.30 | 0.877 |
| 11242 | *atpG* | F_0_F_1_ ATP synthase subunit gamma | 190.49 | 175.90 | 1.08 | 0.987 |
| 11243 | *atpD* | F_0_F_1_ATP synthase subunit beta | 283.20 | 246.60 | 1.15 | 0.996 |
| 11244 | *atpC* | F_0_F_1_ATP synthase subunit epsilon | 36.64 | 32.83 | 1.12 | 1.000 |

**E. Heme biosynthesis (KEGG pathway lic00860) and iron storage**

| LIC ID | Gene | Product | DMC^1^ | IV^1^ | Fold-change | P-adj^2^ |
| --- | --- | --- | --- | --- | --- | --- |
| 11412 |  | coproporphyrinogen III oxidase | 13.16 | 16.77 | -1.28 | 0.974 |
| 13345 | *gltX* | glutamyl-tRNA synthetase | 64.99 | 55.91 | 1.16 | 0.827 |
| **20008** | ***hemA*** | **glutamyl-tRNA reductase** | **30.07** | **189.75** | **-6.25** | **2.68x10^-9^** |
| **20009** | ***hemC*** | **porphobilinogen deaminase** | **30.60** | **114.52** | **-3.70** | **1.82x10^-4^** |
| **20010** | ***hemB*** | **delta-aminolevulinic acid dehydratase** | **26.93** | **61.78** | **-2.27** | **0.036** |
| **20011** | ***hemL*** | **glutamate-1-semialdehyde aminotransferase** | **55.93** | **136.74** | **-2.43** | **0.015** |
| **20012** |  | **histidine kinase sensor protein** | **17.78** | **44.86** | **-2.50** | **0.040** |
| 20013 |  | response regulator | 18.99 | 42.57 | -2.22 | 0.098 |
| **20014** | ***hemE*** | **uroporphyrinogen decarboxylase** | **14.48** | **48.16** | **-3.33** | **0.003** |
| 20015 | *hemN* | coproporphyrinogen III oxidase | 51.78 | 92.19 | -1.79 | 0.341 |
| 20017 | *hemG* | protoporphyrinogen oxidase | 26.12 | 37.71 | -1.45 | 0.611 |
| 20018 | *hemH* | ferrochelatase | 15.81 | 10.11 | 1.56 | 0.497 |
| **20148** | ***hol*** | **heme oxygenase** | **125.93** | **41.11** | **3.06** | **0.003** |

**F. Cobalamin biosynthesis (KEGG pathway lic00860)**

| LIC ID | Gene | Product | DMC^1^ | IV^1^ | Fold-change | P-adj^2^ |
| --- | --- | --- | --- | --- | --- | --- |
| 20119 | *pgmA* | phosphoglycerate mutase | 4.09 | 5.48 | -1.33 | 1.000 |
| 20120 | *cobD* | cobalamin biosynthesis protein | 10.79 | 9.52 | 1.13 | 0.875 |
| 20121 | *cbiP* | histidinol-phosphate aminotransferase and cobyric acid synthase | 46.64 | 46.21 | 1.01 | 0.895 |
| 20122 | *cobU* | cobinamide kinase | 8.17 | 6.61 | 1.23 | 0.954 |
| 20123 |  | hypothetical protein | 28.46 | 26.33 | 1.08 | 0.919 |
| 20124 | *cobB* | cobyrinic acid a,c-diamide synthase | 49.67 | 36.36 | 1.37 | 0.562 |
| 20125 | *cobO* | corrinoid ATP adenosyltransferase | 26.73 | 23.93 | 1.12 | 0.848 |
| 20126 | *cobM* | precorrin-4-methylase | 29.41 | 28.97 | 1.02 | 0.940 |
| 20127 | *cobJ* | precorrin-3 C-17 methylase | 60.26 | 67.95 | -1.12 | 1.000 |
| 20128 | *cbiG* | precorrin methylase | 43.66 | 46.89 | -1.08 | 0.981 |
| 20129 | *cobI* | precorrin-2 C-20 methlytransferase | 26.33 | 32.72 | -1.25 | 0.959 |
| 20130 | *cobL* | precorrin-6y methylase | 75.76 | 72.16 | 1.05 | 0.846 |
| 20131 | *cobH* | precorrin isomerase | 49.80 | 49.42 | 1.01 | 0.940 |
| 20132 | *cbiD* | cobalt-precorrin-6A synthase | 81.13 | 98.68 | -1.22 | 0.981 |
| 20133 | *cobK?* | oxidoreductase with NAD-binding domain | 52.29 | 54.19 | -1.04 | 1.000 |
| 20134 |  | hypothetical protein | 21.68 | 22.82 | -1.05 | 0.969 |
| 20135 | *cbiX* | ferredoxin | 68.19 | 62.31 | 1.09 | 0.840 |
| 13356 | *cobT* | nicotinate-nucleotide-dimethylbenzimidazole phosphoribosyl transferase | 14.66 | 8.64 | 1.70 | 0.539 |
| 13357 | *cobS* | cobalamin 5'-phosphate synthase | 2.75 | 2.19 | 1.26 | 0.827 |
| 13358 | *gpmB* | phosphoglycerate mutase | 2.66 | 0.52 | 5.16 | 0.261 |
| 10204 | *cyoE* | cytochrome C oxidase assembly protein | 24.18 | 26.18 | -1.09 | 1.000 |
| 10205 | *ctaA* | cytochrome oxidase assembly protein | 18.33 | 19.35 | -1.05 | 0.981 |
| 13368 | *cysG* | siroheme synthetase | 12.32 | 17.63 | -1.42 | 0.871 |
| 13369 | *cobA* | uroporphyrinogen-III C-methyltransferase | 21.70 | 23.52 | -1.09 | 1.000 |

**G. Di- and oligo-peptide transporters (KEGG pathway licM00239)**

| LIC ID | Gene | Product | DMC^1^ | IV^1^ | Fold-change | P-adj^2^ |
| --- | --- | --- | --- | --- | --- | --- |
| 11824 | *oppD* | ABC transporter ATP-binding protein | 22.71 | 27.46 | -1.21 | 0.97 |
| 13037 | *lat1* | amino acid transporter | 74.71 | 63.23 | 1.18 | 0.70 |
| 13382 | *dppB* | dipeptide ABC transporter permease | 9.45 | 10.37 | -1.10 | 1.00 |
| 13383 | *dppC* | dipeptide ABC transporter permease | 15.74 | 11.42 | 1.38 | 0.56 |
| 13384 | *dppD* | ABC transporter ATP-binding protein | 11.43 | 6.47 | 1.77 | 0.36 |
| 13385 | *oppF* | ABC transporter ATP-binding protein | 7.47 | 5.95 | 1.26 | 0.79 |

**H. TonB transporters and TonB-dependent receptor proteins**

| LIC ID | Gene | Product | DMC | IV^1^ | Fold-change | P-adj^2^ |
| --- | --- | --- | --- | --- | --- | --- |
| 10889 | *tonB* | TonB | 2.99 | 1.04 | 2.88 | 0.568 |
| 10890 |  | Biopolymer transport protein ExbD/TolR | 1.57 | 1.05 | 1.49 | 0.872 |
| 10891 |  | Biopolymer transport protein ExbD/TolR | 0.68 | 0.00 | - | 0.643 |
| 10892 | *exbB-1* | Biopolymer transport ExbB-like protein | 1.89 | 1.87 | 1.01 | 1 |
| 11621 | *tolQ* | ExbB (TolQ) | 163.85 | 230.13 | -1.41 | 0.634 |
| 11622 |  | Biopolymer transport ExbD-related transmembrane protein | 95.31 | 123.85 | -1.30 | 0.704 |
| 20169 |  | Biopolymer transport protein ExbD/TolR | 4.80 | 3.19 | 1.50 | 0.729 |
| 20170 |  | MotA/TolQ/ExbB proton channel family | 6.68 | 5.13 | 1.30 | 0.901 |
| 20216 | *exbB-2* | ExbB | 78.95 | 89.18 | -1.12 | 0.755 |
| 20217 | *exbD* | ExbD | 47.64 | 43.94 | 1.08 | 0.981 |
| 20218 |  | TonB | 41.44 | 55.70 | -1.35 | 0.788 |
| 10714 | *smc* | TB-DR | 114.18 | 50.82 | 2.25 | 0.159 |
| 10881 |  | TB-DR | 4.40 | 2.12 | 2.07 | 0.822 |
| 10896 | *fecA* | TB-DR | 3.15 | 6.49 | 2.08 | 0.628 |
| **10964** | *phuR* | **TB-DR (hemin receptor)** | **25.33** | **7.33** | **3.46** | **0.022** |
| 10998 |  | Hypothetical protein (putative TB-DR) | 11.59 | 10.86 | 1.07 | 0.848 |
| 11345 |  | TB-DR | 36.45 | 26.09 | 1.40 | 0.700 |
| **11694** |  | **TB-DR** | **52.49** | **3.53** | **14.85** | **2.2x10^-13^** |
| 12374 | *btuB* | TB-DR (Vitamin B12) | 28.42 | 17.85 | 1.59 | 0.384 |
| 12898 |  | Hypothetical protein (putative TB-DR) | 6.32 | 7.85 | -1.25 | 0.888 |
| 12998 |  | Hypothetical protein (putative TB-DR) | 5.84 | 4.71 | 1.24 | 0.934 |
| 20151 |  | TB-DR | 14.22 | 27.36 | -1.92 | 0.269 |
| 20214 |  | Hypothetical protein (putative TB-DR) | 99.03 | 151.85 | -1.54 | 0.481 |

**I. Classical stress response genes**

| LIC ID | Gene | Product | DMC^1^ | IV^1^ | Fold-change | P-adj^2^ |
| --- | --- | --- | --- | --- | --- | --- |
| 10523 | *dnaJ* | Chaperone protein DnaJ | 390.51 | 615.74 | 1.58 | 0.587 |
| 10524 | *dnaK* | Molecular chaperone DnaK | 1,741.08 | 3,472.34 | 1.99 | 0.711 |
| 10525 | *grpE* | Heat shock protein GrpE | 288.54 | 678.20 | 2.35 | 0.227 |
| 10526 | *hrcA* | Heat-inducible transcriptional repressor | 224.78 | 371.17 | 1.65 | 0.490 |
| 11335 | *groEL* | Chaperonin GroEL | 2,577.19 | 958.88 | 2.69 | 0.328 |
| 11336 | *groES* | Chaperonin GroES | 488.12 | 229.07 | 2.13 | 0.224 |
| 20044 | *htpG* | Molecular chaperone HtpG (Hsp90 ortholog) | 340.90 | 284.89 | 1.20 | 0.978 |

**J. Cell shape and membrane fluidity**

| LIC ID | Gene | Product | DMC^1^ | IV^1^ | Fold-change | P-adj^2^ |
| --- | --- | --- | --- | --- | --- | --- |
| **13053** | ***desA*** | **Fatty acid desaturase** | **124.22** | **18.49** | **6.72** | **2.98x10^-11^** |
| 13182 | *ugpQ* | Glycerophosphoryl diester phosphodiesterase (cytosol) | 15.68 | 11.77 | 1.33 | 0.752 |
| 10293 | *glpQ* | Glycerophosphoryl diester phosphodiesterase (OM) | 22.51 | 14.01 | 1.61 | 0.779 |
| 11806 | *bolA* | BolA-like protein | 0.41 | 1.89 | -4.55 | 0.776 |

**K. Known or putative adhesins**

| LIC ID | Gene | Product | DMC^1^ | IV^1^ | Fold-change | P-adj^2^ |
| --- | --- | --- | --- | --- | --- | --- |
| 10258 | *lsa66* | Hypothetical protein (Lsa66) | 86.68 | 55.00 | 1.58 | 0.529 |
| 10314 | *lsa63* | Hypothetical protein (Lsa63) | 302.63 | 194.04 | 1.56 | 0.577 |
| 10368 | *lsa21* | Lipoprotein (Lsa21) | 7.55 | 11.85 | -1.56 | 0.539 |
| 10464 | *ligB* | Bacterial Ig-like (Big) repeat domain protein 3 | 3,447.50 | 1,125.45 | 3.06 | 0.254 |
| **10465** | ***ligA*** | **LigA Bacterial Ig-like (Big) repeat domain protein 1** | **2,633.11** | **576.22** | **4.57** | **0.0203** |
| 10997 | *lenB* | Hypothetical protein (LenB) | 6.27 | 4.40 | 1.43 | 0.848 |
| 11087 | *lsa30* | Lipoprotein (Lsa30) | 1.96 | 1.53 | 1.29 | 1 |
| 11352 | *lipL32* | Hypothetical protein (LipL32) | 2,625.31 | 2,744.30 | -1.04 | 0.985 |
| 11469 | *lsa20* | Hypothetical protein (Lsa20) | 8.38 | 19.58 | -1.23 | 0.432 |
| 11834 | *lsa33* | Lipoprotein (Lsa33) | 38.27 | 46.34 | -1.20 | 0.735 |
| 11947 | *lcpA* | Leptospiral complement regulator-acquiring protein A | 50.79 | 50.77 | 1.00 | 1 |
| **12099** | ***lipL53*** | **Hypothetical protein Lsa53/LipL53** | **258.43** | **13.25** | **19.50** | **1.03E-26** |
| 12253 | *lsa25* | Lipoprotein (Lsa25) | 19.34 | 26.79 | -1.39 | 0.685 |
| **12263** | ***ompL37*** | **Hypothetical protein (OmpL37)** | **52.26** | **19.58** | **2.67** | **8.81E-03** |
| 12315 | *lenD* | Liporotein (LenD) | 42.74 | 24.06 | 1.78 | 0.486 |
| 12690 | *Lp95* | Lipoprotein (Lp95) | 7.41 | 12.14 | -1.64 | 0.464 |
| 12895 | *lsa27* | Lipoprotein (Lsa27) | 33.91 | 30.88 | 1.10 | 0.989 |
| 12906 | *lenA* | LenA/Lsa24/LfhA Endostatin-like protein A | 3.08 | 1.60 | 1.93 | 0.901 |
| 12976 |  | Lipoprotein | 44.45 | 49.17 | -1.11 | 1 |
| 13006 | *lenC* | Lipoprotein (LenC) | 29.64 | 14.14 | 2.10 | 0.300 |
| 13050 | *ompL47* | Hypothetical protein (OmpL47) | 567.24 | 457.67 | 1.24 | 0.886 |
| 13248 | *lenF* | Lipoprotein (LenF) | 22.36 | 16.23 | 1.38 | 0.875 |
| 13467 | *lenE* | Hypothetical protein (LenE) | 17.26 | 18.24 | 0.95 | 1 |

Differentially-expressed genes are highlighted in **bold**.

^1^ Mean values per gene from three biological replicates in either DMC or *in vitro* (IV) conditions (see Table S2).

^2^ Adjusted P-value (see Table S2).
