# Supplementary material for: A Model System for Studying the Transcriptomic and Physiological Changes Associated with Mammalian Host-Adaptation by Leptospira interrogans Serovar Copenhageni
Source: PLoS Pathog. 2014 Mar 13;10(3):e1004004. doi: 10.1371/journal.ppat.1004004 (PMC3953431; doi:10.1371/journal.ppat.1004004)
Supplement: Table S5 — Redox-relevant proteins encoded within the genomes of the pathogenic spirochetes Treponema pallidum , Borrelia burgdorferi and L. interrogans sv. Copenhageni. (DOCX) [file ppat.1004004.s009.docx]

**Table S5. Redox-relevant proteins encoded within the genomes of the pathogenic spirochetes *Treponema pallidum*, *Borrelia burgdorferi* and *L. interrogans* sv. Copenhageni.**

| *Treponema pallidum^1^* | *Borrelia burgdorferi^1^* | Protein Family | *Leptospira interrogans* | | | | | | | | | |  |
| --- | --- | --- | --- | --- | --- | --- | --- | --- | --- | --- | --- | --- | --- |
|  |  |  | LIC ID | | Gene | DMC^3^ | | IV^3^ | | Fold-change | | P-adj^4^ | |
| Yes | No | Peroxiredoxin (AhpC) | **11219** | ***ahpC*** | | | **859.87** | **144.23** | **5.96** | | **7.29 x 10^-7^** | | |
| No | No | Catalase | 12032 | *katE* | | | 1363.79 | 761.75 | 1.79 | | 0.681 | | |
| Yes | Yes | Thioredoxin reductase (TxrR) | 11470 | *trxB (trxR)* | | | 43.19 | 67.09 | -1.56 | | 0.328 | | |
| Yes (2)^2^ | Yes (1)^2^ | Thioredoxin (2)^2^ | 11978 | *trxA* | | | 144.99 | 131.78 | 1.10 | | 0.977 | | |
|  |  |  | 10278 |  | | | 4.42 | 3.09 | 1.43 | | 0.848 | | |
|  |  | Polyketide synthase (TrxR-like) | **10149** | ***frnE*** | | | **23.76** | **4.86** | **4.89** | | **1.08E-02** | | |
| No | No | Thiol peroxidase | 12765 | *tpx* | | | 16.07 | 23.17 | -1.45 | | 0.700 | | |
| No | No | Glutathione peroxidase (2)^2^ | 12648 | *btuE* | | | 52.75 | 20.79 | 2.54 | | 0.086 | | |
|  |  |  | 13442 | *gpo* | | | 50.35 | 17.38 | 2.90 | | 0.054 | | |
| No | No | Glutaredoxin-related protein | 11809 |  | | | 30.32 | 39.88 | -1.32 | | 0.586 | | |
| No | No | Glutathione S-transferase GST (3)^2^ | 10807 | *gst-1* | | | 44.45 | 88.64 | -2.00 | | 0.063 | | |
|  |  |  | 11363 | *gst-2* | | | 14.27 | 9.50 | 1.50 | | 0.640 | | |
|  |  |  | **12981** | ***gst-3*** | | | **10.16** | **2.54** | **4.00** | | **0.026** | | |
| No | No | γ-Glutamate-cysteine ligase | 11812 | *gshA* | | | 36.10 | 51.23 | -1.43 | | 0.679 | | |
| Yes | Yes | DNA-binding stress protein (Dps) | 10606 | *dps* | | | 63.09 | 48.96 | 1.29 | | 0.909 | | |
| No | No | Cytochrome c peroxidase (3)^2^ | **12927** |  | | | **148.91** | **35.23** | **4.23** | | **1.04 x 10^-5^** | | |
|  |  |  | 11088 |  | | | 1.49 | 2.41 | -1.61 | | 0.740 | | |
|  |  |  | 10972 |  | | | 10.47 | 9.42 | 1.11 | | 0.987 | | |
| Yes (1)^2^ | No | Bacterioferritin | 11310 | *bfr* | | | 177.82 | 202.40 | -1.14 | | 0.848 | | |
| No | No | Bacterioferritin co-migratory protein (2)^2^ | 20093 | *bcp-1* | | | 45.85 | 52.29 | -1.44 | | 0.852 | | |
|  |  |  | 10732 | *bcp-2* | | | 23.34 | 29.89 | -1.28 | | 0.629 | | |
| No | No | Ferredoxin | 13258 | *fdx* | | | 6.71 | 9.96 | -1.49 | | 0.646 | | |
| No | No | Bacterioferritin-associated ferredoxin | 13209 |  | | | 40.00 | 21.81 | 1.83 | | 0.185 | | |
| Yes | No | Superoxide dismutase (SOD) | No |  | | |  |  |  | |  | | |
| No | Yes | Superoxide reductase | No |  | | |  |  |  | |  | | |
| No | No | OxyR | No |  | | |  |  |  | |  | | |
| No | No | SoxR | No |  | | |  |  |  | |  | | |

^1^ Based on previously published report by Parsonage *et al.* [67]

^2^ Value in parentheses indicates number of paralogs identified in the respective genome.

^3^ Mean values per gene from three biological replicates in either DMC or *in vitro* (IV) conditions (see Table S2).

^4^ Adjusted P-value (see Table S2).
